# Contribution of plasma membrane lipid domains to red blood cell (re)shaping

**DOI:** 10.1038/s41598-017-04388-z

**Published:** 2017-06-27

**Authors:** C. Leonard, L. Conrard, M. Guthmann, H. Pollet, M. Carquin, C. Vermylen, P. Gailly, P. Van Der Smissen, M. P. Mingeot-Leclercq, D. Tyteca

**Affiliations:** 1 FACM Unit, Louvain Drug Research Institute & Université catholique de Louvain, 1200 Brussels, Belgium; 2 CELL Unit, de Duve Institute & Université catholique de Louvain, 1200 Brussels, Belgium; 3 PEDI Unit, Institut de Recherche expérimentale et clinique & Université catholique de Louvain, 1200 Brussels, Belgium; 4 CEMO Unit, Institute of Neuroscience & Université catholique de Louvain, 1200 Brussels, Belgium

## Abstract

Although lipid domains have been evidenced in several living cell plasma membranes, their roles remain largely unclear. We here investigated whether they could contribute to function-associated cell (re)shaping. To address this question, we used erythrocytes as cellular model since they (i) exhibit a specific biconcave shape, allowing for reversible deformation in blood circulation, which is lost by membrane vesiculation upon aging; and (ii) display at their outer plasma membrane leaflet two types of submicrometric domains differently enriched in cholesterol and sphingomyelin. We here reveal the specific association of cholesterol- and sphingomyelin-enriched domains with distinct curvature areas of the erythrocyte biconcave membrane. Upon erythrocyte deformation, cholesterol-enriched domains gathered in high curvature areas. In contrast, sphingomyelin-enriched domains increased in abundance upon calcium efflux during shape restoration. Upon erythrocyte storage at 4 °C (to mimick aging), lipid domains appeared as specific vesiculation sites. Altogether, our data indicate that lipid domains could contribute to erythrocyte function-associated (re)shaping.

## Introduction

The acknowledgment of lipid heterogeneous lateral distribution in plasma membrane (PM) has changed our perception of their role, from simple cellular compartmentation structures to active participants in cellular functions^1^. In the 90’s, Simons and collaborators proposed that cholesterol (chol) and sphingolipids (SLs) can cluster into nanometric (10–200 nm), unstable (sec) assemblies, called the lipid rafts^2^. From that time forward, the implication of lipid rafts in membrane sorting^3^, signal transduction^4^ and membrane trafficking^5^ has been highly discussed, but spatial and temporal features of such domains make direct demonstrations challenging to provide. Besides rafts, direct lines of evidence for larger (submicrometric) and more stable (min) domains, enriched or not in chol and SLs, were first reported in artificial membranes^6–8^ then in fixed cells^9–11^ and more recently in living cells, including red blood cells (RBCs)^12–15^.

RBC is the most simple and best characterized human cell, whose only structural components are a PM linked to an underlying cytoskeleton. Furthermore, RBC featureless membrane and absence of lipid trafficking facilitate the investigation of PM lateral heterogeneity. During the past years, we examined the organization of abundant lipids of the RBC outer PM leaflet, sphingomyelin (SM) and chol, by confocal vital imaging of RBCs spread onto poly-L-lysine (PLL)-coated coverslips^12–15^. We first inserted at the RBC surface BODIPY analogs of SM. Whereas this approach allowed us to evidence submicrometric domains (Supplementary Fig. 1Aa), it nevertheless presents the limitation that PM-inserted probes can differentially partition as compared to endogenous lipids, depending on membrane lipid composition and on the fluorophore^16^. Thus, to further explore relevance of fluorescent domains for endogenous lipids, we used mCherry-non-toxic parts of Toxins, *i.e*. the minimal fragment of the Lysenin Toxin responsible for specific SM binding^11^ and the D4 Theta domain as the minimal Toxin fragment able to bind to chol with high affinity without causing lysis^17^. We verified that both Toxin fragments (hereafter referred as Lysenin* & Theta*) are specific, non-toxic, sensitive and quantitative probes of SM and chol accessible at the outer PM leaflet of living RBCs^12, 14^. We then provided evidence for SM- and chol-enriched domains similar to those observed upon trace insertion of BODIPY-SM (Supplementary Fig. 1Ab,c). Importantly, BODIPY-SM and Lysenin* perfectly colocalize (Supplementary Fig. 1Ba). SM- and chol-enriched domains (i) exhibit a peak of abundance at 20 °C and are maintained at 37 °C, although to a lower extent; (ii) are similarly increased upon membrane:cytoskeleton uncoupling at 4.1 R complexes (Supplementary Fig. 1Cc,g); and (iii) show a reciprocal dependence as revealed by disappearance of domains upon specific chol or SM depletion (Supplementary Fig. 1Cb,f). In contrast, SM- and chol-enriched domains exhibit a differential response to increased membrane tension (Supplementary Fig. 1Cd,h) and are only partially spatially colocalized (Supplementary Fig. 1Bb). Altogether, these data indicate the coexistence of two types of domains, one enriched in SM/chol *vs* another enriched in chol mainly. However, despite evidence for PM submicrometric lipid domains, their specific functions remain largely unknown^18^.

PMs have evolved in a wide range of function-associated shapes and the mechanisms underlying membrane complex architecture shaping and remodelling in relation with cellular functions generate broad interest^19, 20^. The RBC is one typical example of cells exhibiting specific (re)shaping in relation to their functions. Indeed, RBC exhibits a specific biconcave shape in circulation, which results in high area-to-volume ratio with a ~40% surface excess as compared to a sphere of the same volume. This specific shape both ensures fast oxygen and carbon dioxide exchanges between the RBC interior and its environment but also decreases the forces that have to be applied to deform the membrane as compared to a spherical shape^21^. Such deformability is required when RBC passes through the microvasculature to deliver oxygen to the tissues, and is further tested for quality control when it squeezes through the very narrow pores of the spleen sinusoids. At the end of its 120-day lifetime, biconcavity and deformability are lost due to local membrane vesiculation, leading to RBC splenic entrapment and removal from blood circulation^22^. Whether and how submicrometric lipid domains contribute to RBC (re)shaping is currently not known. Maintenance of the specific RBC biconcave shape^23^, global membrane shape changes upon deformation^24^ and local membrane vesiculation upon aging^25^ are generally attributed to the dynamic, strongly membrane-anchored cytoskeleton. RBC (re)shaping also implies tight volume regulation by ion exchanges. For instance, following RBC membrane mechanical stress, intracellular calcium (Ca^2+^) concentration transiently increases, followed by the secondary activation of the Gardos channel leading to transient volume decrease^26–28^.

We here took benefit from the RBC wide range of function-related (re)shaping processes to investigate the potential role of submicrometric lipid domains in cell shape control. To this end, we probed by vital imaging the lateral distribution of chol and SM (using either specific Toxin fragments or trace insertion of BODIPY-SM as previously^12, 14^) in relation with: (i) membrane biconcavity of resting RBC; (ii) membrane curvature changes and Ca^2+^ exchanges upon mechanical stretching of healthy RBCs or in elliptocytes, a RBC model of impaired shape^22^; and (iii) membrane vesiculation upon RBC aging. Our results revealed that, although they were not essential to the maintenance of RBC biconcavity, chol- and SM-enriched domains specifically associated with high and low curvature areas of the biconcave RBC membrane. Upon reshaping, chol-enriched domains gathered in areas of increased curvature, as revealed in elliptocytes and healthy RBC stretching experiments. In contrast, SM-enriched domains increased in abundance along with Ca^2+^ efflux during subsequent shape and volume restoration. Finally, both domains were identified as specific sites for membrane vesiculation. Altogether, our results revealed the contribution of lipid domains to erythrocyte function-associated (re)shaping.

## Results

### Distinct topographic distribution of chol- and SM-enriched domains in both spread and suspended RBCs

**Figure 1:**
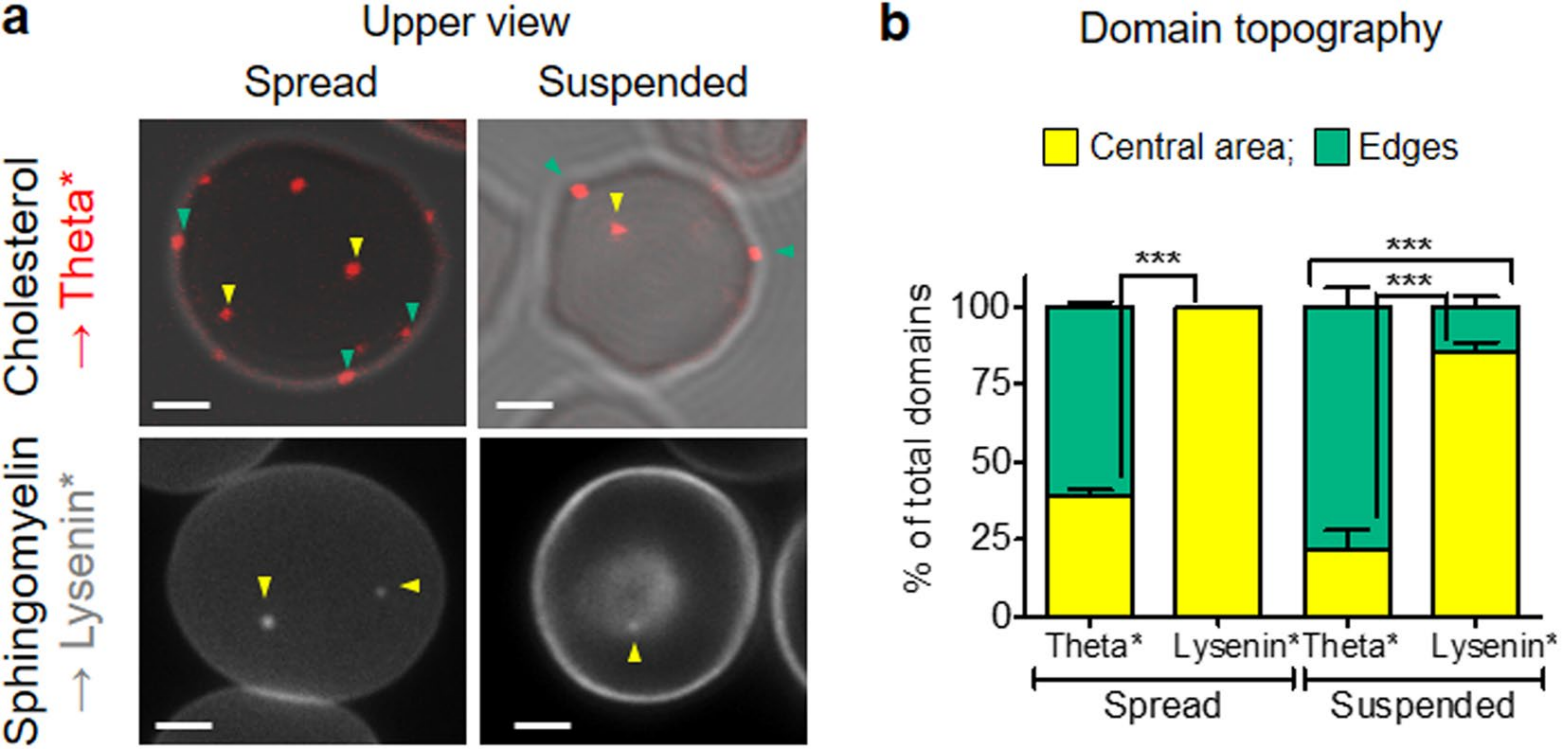
Distinct topographic distribution of chol- and SM-enriched domains in both spread and suspended RBCs. Fresh healthy RBCs were labelled in suspension with Theta* (*red; chol*) or Lysenin* *(grey; SM)*, washed and then either spread onto poly-L-lysine (PLL)-coated coverslips *(spread)* or left in suspension *(suspended)* and directly visualized by vital confocal/fluorescence microscopy. (**a**) Representative imaging. *Yellow* and *green arrowheads* point to the central area and the edges of the RBC membrane, respectively. Representative of 3 experiments, s*cale bars* 2 µm. (**b**) Quantification of domain association (number by hemi-RBC expressed as percentage of total domains) with the central area (*yellow*) and the edges (*green*) of the RBC membrane. Results are means ± SEM of 40–150 RBCs from three independent experiments. Statistical significance was tested with two-sample t-tests.

We first analyzed lipid domain distribution on Theta* (*red*) or Lysenin* (*grey*)-labeled RBCs spread onto poly-L-lysine (PLL)-coated coverslips. We observed that the submicrometric domains respectively enriched in chol or SM were not randomly distributed (Fig. 1a, *spread upper view*); while chol-enriched domains were associated with both the center of the RBC membrane (*yellow arrowheads*) and the edges (*green arrowheads*), those enriched in SM were restricted to the center of the membrane (quantification at Fig. 1b, *spread*). Although RBC spreading is an easy system for lipid domain imaging (preventing RBC from moving during image acquisition and allowing to analyze the major domain population in one image due to RBC flattening), it was nevertheless crucial to ask about the influence of RBC spreading on intrinsic lipid domain topography.

Therefore, we used an alternative imaging system in which RBCs were laid down in medium, giving rise to suspended, non-spread, RBCs (Fig. 1a, *suspended vs spread*). Suspended RBCs exposed similar domain abundance as compared to spread RBCs, as revealed by full projection (compare Supplementary Fig. 2a with Fig. 1a, *spread*). Importantly, the respective preferential association of chol- and SM-enriched domains with the edges (*green arrowheads*) and the center (*yellow arrowheads*) of the PM was confirmed in this observation system, despite the slightly higher proportion of both chol- and SM-enriched domains associated with the edges (Fig. 1a; quantification at Fig. 1b, *suspended*). We thus concluded to the preferential association of chol- and SM-enriched domains with the edges and the center of RBC membrane, respectively, as revealed in both spread and suspended systems.

### Specific association of chol- and SM-enriched domains with high and low curvature areas of the biconcave RBC membrane, respectively

**Figure 2:**
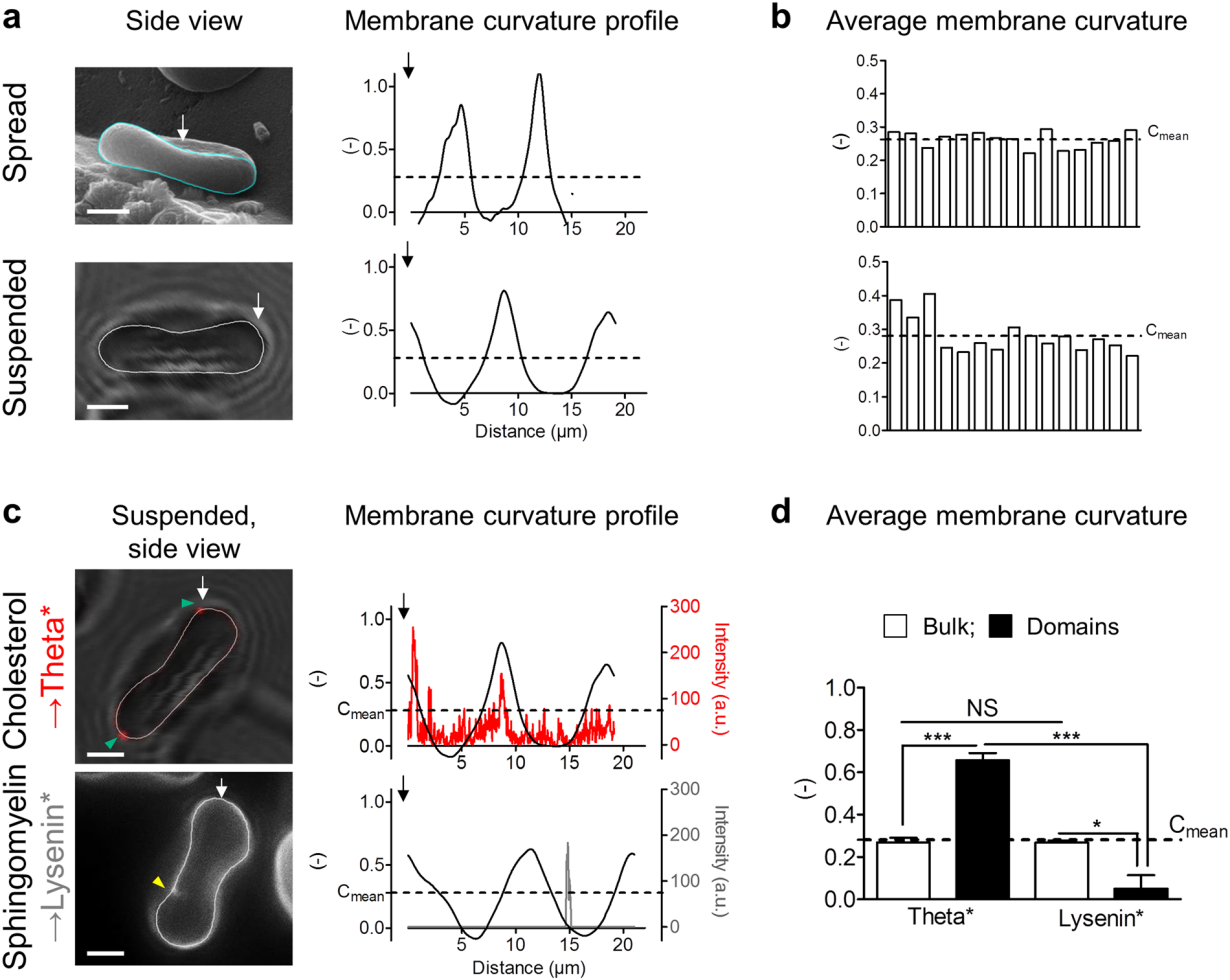
Specific association of chol- and SM-enriched domains with high and low curvature areas of the RBC biconcave membrane, respectively. Fresh healthy RBCs were labelled or not (**a,b**) as in Fig. 1 (**c,d**), washed, then either spread onto PLL-coated coverslips *(spread)* and observed by scanning electron microscopy (SEM), or left in suspension *(suspended)* and visualized by vital confocal/fluorescence microscopy. (**a**) Representative imaging of side viewed unlabelled RBCs *(left panels)* and membrane curvature profiles along paths indicated at *left* by *cyan or white curved lines* (*right panels)*. Profiles are going counterclockwise from starting points indicated by *white arrows*. Representative of three independent experiments, *scale bars* 2 µm. (**b**) Quantification of average side viewed membrane curvature(*C*_*mean*_). *Dashed lines* represent the average membrane curvature of 15 side viewed RBCs (0.26 for *spread* and 0.28 for *suspended RBCs*). (**c**) Representative imaging (*left panels, yellow* and *green arrowheads* point to the central area and the edges of the RBC membrane, respectively*)* and profiles comparing Theta* or Lysenin* intensity (*red* or *grey*) and membrane curvature (*black*; *right panels)*. Profiles along paths indicated in *left panel* by *white curved lines* on side viewed suspended RBCs (counterclockwise from starting point indicated by *white arrows*). *Dashed lines* (*C*_*mean*_) represent the average membrane curvature calculated in (**b**). Representative imaging and profiles of three independent experiments, s*cale bars* 2 µm. (**d**) Quantification of average curvature values for bulk membrane (membrane without domains, *open bars*) and Theta*- or Lysenin*-enriched domains (*filled bars*), calculated on profiles from side viewed suspended RBCs. *Dashed lines* represent the average curvature value calculated in (**b**). Results are means ± SEM of 4–10 RBCs from three independent experiments. Statistical significance was tested with one-way ANOVA followed by Tukey’s post-hoc test.

The distinct topographic distribution of chol- and SM-enriched domains raised the hypothesis of their association with different membrane curvature areas, a feature obviously distinguishing RBC membrane center and edges and resulting from its singular biconcave shape. To investigate this question we turned to RBC observation in side view. We first explored by scanning electron microscopy (SEM) the membrane curvature of spread unlabelled RBCs in side view (Fig. 2a, *spread*) and calculated on a series of membrane curvature profiles the average membrane curvature value (Fig. 2a and b, *C*_*mean*_). This value was defined as the threshold between low and high membrane curvature areas in side-viewed spread RBCs. Using this value, we confirmed on side-viewed membrane curvature profiles that the edges were associated to high curvature areas (>*C*_*mean*_, referred as *HC* in figures), while the center corresponded to low curvature area (<*C*_*mean*_, referred as *LC* in figures). To further evaluate the impact of RBC spreading on side-viewed membrane curvature, we applied the same approach on suspended RBCs using vital confocal imaging. Indeed, this in suspension imaging system allows RBCs to lay down either on their flat side or their edges, and thus observation in either upper or side view (Fig. 2a and b, *suspended*). The calculated threshold value and curvature profiles were very close to those of spread RBCs, suggesting that, in our hands, RBC spreading did not significantly impact biconcavity. Furthermore, like in spread RBCs, the edges were associated to high curvature areas (>*C*_*mean*_), while the center of the RBC membrane corresponded to low curvature area (<*C*_*mean*_).

To next examine whether chol- and SM-enriched domains could associate with distinct membrane curvature areas, we compared membrane curvature profiles with Theta* or Lysenin* intensity profiles on suspended RBCs observed in side view by vital imaging. Chol- and SM-enriched domains respectively associated with high (>*C*_*mean*_) and low (<*C*_*mean*_) curvature areas, as revealed by profile analysis (Fig. 2c and gallery of profiles in Supplementary Figs 3 and 4), and exposed average curvature values respectively higher and lower than the one of the bulk membrane (membrane without domains) and *C*_*mean*_ (Fig. 2d). Furthermore, we asked if these domains were not only associated to, but also involved in, RBC specific biconcave curvature. We therefore abrogated both chol- and SM-enriched domains by treatment with methyl-ß-cyclodextrin (mßCD) as previously^12, 14^, but did not observe any impact on RBC biconcavity (Supplementary Fig. 5).

Our results thus suggested that, while chol- and SM- enriched domains were associated to distinct membrane curvature areas of the biconcave RBC, they were not involved in the maintenance of this specific shape.

### Preferential association of chol-enriched domains with increased curvature areas in the rim of elliptocytes

**Figure 3:**
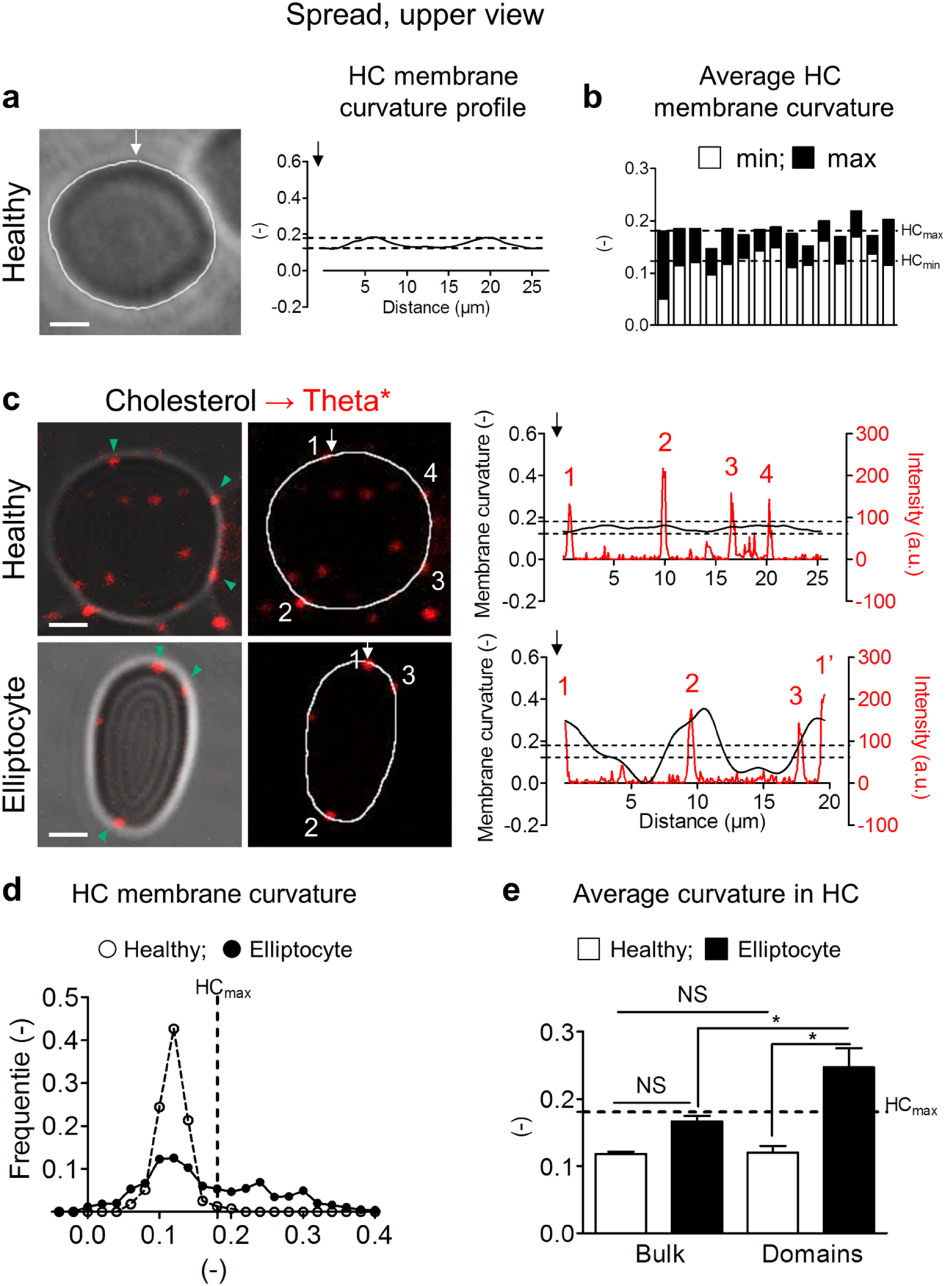
Preferential association of chol-enriched domains with increased curvature areas in the rim of elliptocytes. Fresh healthy and elliptocytotic RBCs were labelled (**c–e**) or not (**a,b**) with Theta*, washed, spread and observed in upper view as in Fig. 1. (**a**) Representative phase contrast image (*left panel)* and membrane curvature profile along path indicated in *left panel* by *white curved line* (*right panel)*. Profile is going counterclockwise from starting point indicated by *white arrow*. Representative of three independent experiments, s*cale bar* 2 µm. (**b**) Quantification of minimum and maximum membrane curvature values. *Dashed lines* represent the average minimum and maximum curvature values of 15 side viewed RBCs (0.12 for *HC*_*min*_ and 0.18 for *HC*_*max*_). (**c**) Representative vital confocal imaging of Theta*-labelled RBCs (*left & central panels; green arrowheads* indicate domains in HC membrane areas) and relation between Theta* intensity (*red*) and membrane curvature (*black*) on profiles along paths indicated *in central panel* by *white curved lines* starting by *white arrows (right panel)*. Domains (*red numbers*) are labelled with *white numbers* in *central panel; dashed lines* represent HC_min_ and HC_max_ calculated in (**b**). Representative of four independent experiments, s*cale bars* 2 µm. (**d**,**e**) Quantification of curvature value distribution of HC membrane (**d**) and average curvature values of HC bulk and domains (**e**) calculated on profiles of healthy RBCs (*open symbols and bars*) and elliptocytes (*black symbols and bars)*. Results are means ± SEM of 4–10 RBCs from two independent experiments. Statistical significance was tested with one-way ANOVA followed by Tukey’s post-hoc test.

The different association of chol- and SM-enriched domains with distinct membrane curvature areas raised the question of their implication in membrane curvature changes required for RBC reshaping. As a model of affected membrane curvature, we used RBCs from a patient suffering from elliptocytosis, a genetic disease resulting from impaired RBC cytoskeleton and leading to RBC shape changes^29^. Regarding membrane curvature, suspended elliptocytes exhibited no changes in side-viewed biconcave membrane (compare Supplementary Fig. 6a with Fig. 2c) but affected upper-viewed membrane edges/rim as compared to healthy RBCs (compare Supplementary Fig. 6b with Fig. 1a, right panel). To quantify these changes in elliptocyte rim, we therefore analysed membrane curvature of RBCs in upper view. We determined membrane curvature profiles on a series of upper-viewed healthy RBC membranes (Fig. 3a), and calculated the average minimum (Fig. 3b, *HC*_*min*_) and maximum curvatures (*HC*_*max*_) as threshold values to determine decreased (<*HC*_*min*_) and increased (>*HC*_*max*_) curvature areas in the rim of elliptocytes *vs* discoid healthy RBCs (Fig. 3c). Change in curvature associated with elliptocytosis was confirmed by the wider distribution of membrane curvature values in elliptocytes *vs* healthy RBCs (Fig. 3d).

We then used the HC_max_ value to explore whether membrane curvature increase was associated to changes in lipid domain distribution in the rim of elliptocytes *vs* healthy RBCs. Profile analyses (Fig. 3c) and resulting average membrane curvature values (Fig. 3e) indicated that chol-enriched domains were associated with increased curvature areas (>*HC*_*max*_) of the elliptocyte rim. In contrast, no impact was observed on SM-enriched domain topography, still restricted to the center, and no specific SM enrichment was observed in areas of increased curvature of the rim (Supplementary Fig. 7). Thus, chol-, but not SM-, enriched domains specifically associated with increased curvature areas generated in the rim of elliptocytes.

### Specific recruitment of chol-enriched domains in increased curvature areas of the RBC rim upon stretching

**Figure 4:**
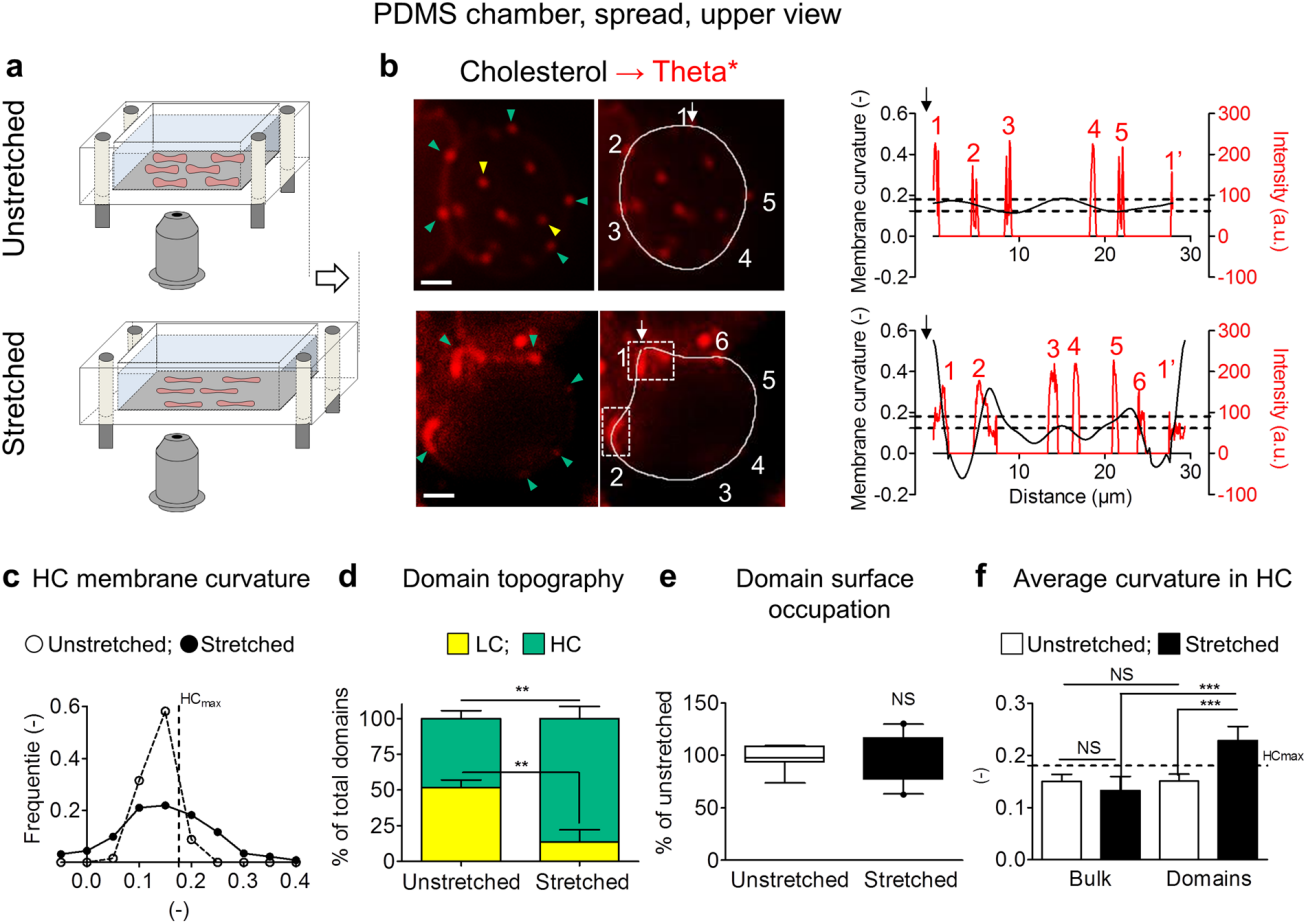
Recruitment of chol-enriched domains in increased curvature areas of the RBC rim upon stretching. Fresh healthy RBCs were labelled with Theta*, washed and spread onto PLL-coated polydimethylsiloxane (PDMS) chamber and directly visualized by vital fluorescence microscopy before (*unstretched*) and after stretching (*stretched*). (**a**) Schematic representation of the stretching system. (**b**) Representative imaging (*left & central panels*) and comparison of Theta* intensity (*red*) and membrane curvature (*black*) profiles (*right panel*). Y*ellow* and *green arrowheads* indicate LC and HC membrane areas, respectively. Profiles are done along paths indicated *in central panel* by *white curved lines* starting by *white arrows*; domains (*red numbers*) are labelled with *white numbers* in *central panel*; *dashed lines* represent HC_min_ and HC_max_. Theta*-labelled domains associated with increased curvature (>HC_max_) are boxed in *central panel*. Representative of two independent experiments, s*cale bars* 2 µm. (**c**) Quantification of curvature value distribution of HC membrane calculated on profiles of unstretched (*open symbols)* and stretched RBCs (*black symbols*). (**d**,**e**) Quantification of domain topography (d; number by hemi-RBC, expressed as percentage of total domains) and surface occupation (e; total domain area by hemi-RBC area, expressed as percentage of unstretched). (**f**) Quantification of average curvature values of HC bulk membrane and domains. Results are means ± SEM of 13–18 RBCs from two independent experiments. Statistical significance was tested with two-sample t-tests, except one-way ANOVA followed by Tukey’s post-hoc test in f.

As chol-enriched domains specifically associated with increased curvature areas created in the rim of elliptocytes, we asked whether these domains could participate in membrane curvature changes during RBC deformation by stretching. Theta*-labeled healthy RBCs were spread onto PLL-coated polydimethylsiloxane (PDMS) chambers and observed in their resting state (Fig. 4a, *unstretched*) or upon chamber stretching (*stretched*). RBC stretching induced visible changes in membrane curvature of upper-viewed RBCs (Fig. 4b; quantification in Fig. 4c) and an increased association of chol-enriched domains with the cell edges (*green arrowheads* at Fig. 4b; quantification in Fig. 4d). Total occupation of the RBC surface by chol-enriched domains (Fig. 4e) and associated total Theta* intensity (Supplementary Fig. 8) were not modified upon stretching. This could indicate that domains were neither formed nor lost during stretching, suggesting that chol-enriched domain topographic changes are related to domain rearrangement. Furthermore, chol-enriched domains specifically gathered in areas of increased curvature of the membrane rim (white dashed boxes at Fig. 4b, >*HC*_*max*_), which was confirmed by the increased average curvature of chol-enriched domains after stretching, above the bulk average curvature and HC_max_ (Fig. 4f & gallery of profiles in Supplementary Fig. 9). In contrast, no recruitment of SM-enriched domains in areas of modified curvature was observed (data not shown). Altogether, these results indicated the specific recruitment of chol-, but not SM-, enriched domains in areas of increased curvature of the membrane rim upon RBC deformation.

### Loss of high curvature areas in elliptocytes and impairment of healthy RBC deformability upon chol-enriched domain abrogation

**Figure 5:**
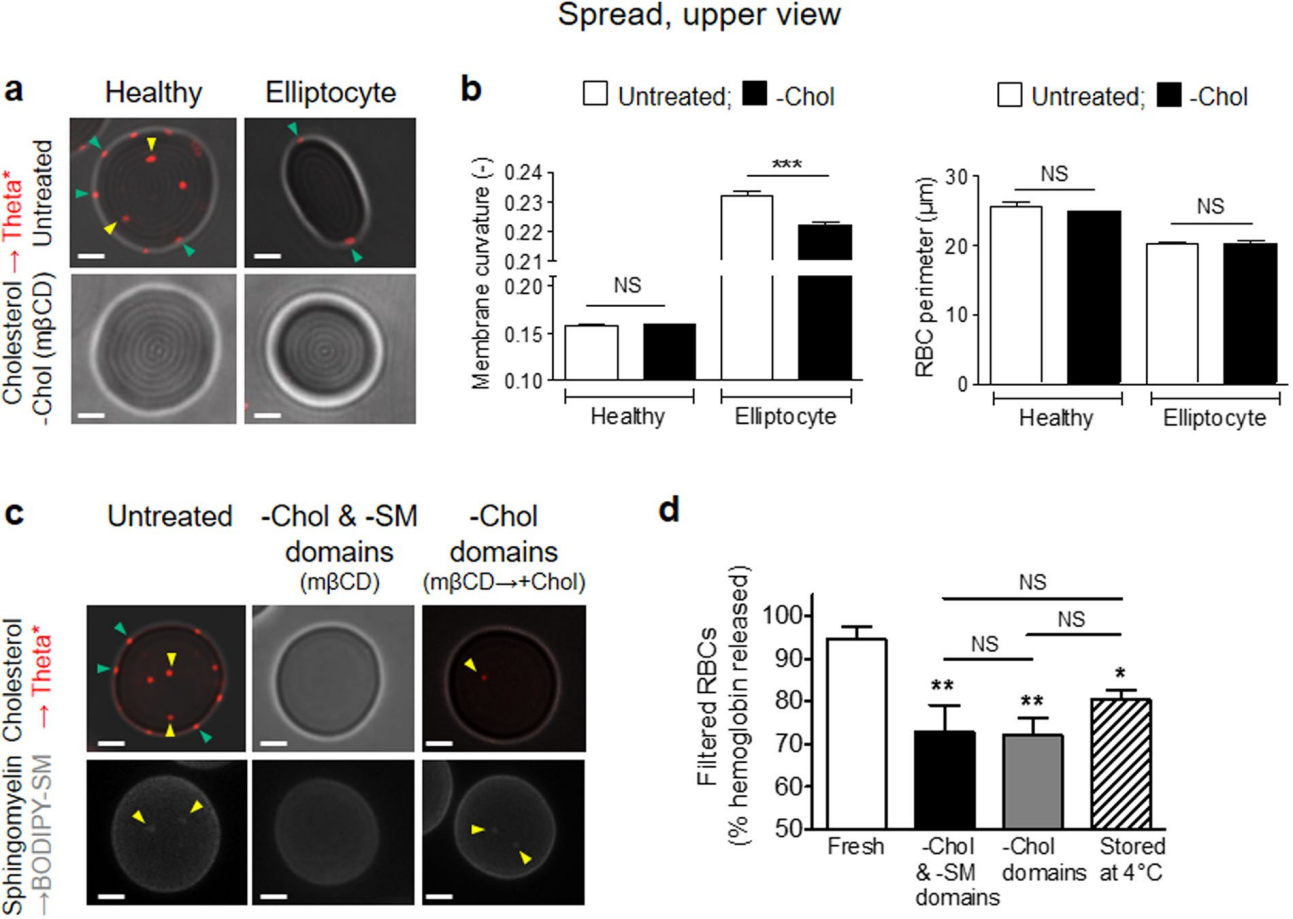
Loss of high curvature areas in elliptocytes and impairment of healthy RBC deformability upon chol-enriched domain abrogation. (**a,b**) Relation between lipid domains and RBC curvature. Fresh healthy and elliptocytotic RBCs were either left untreated (*untreated*) or incubated with methyl-ß-cyclodextrin (mßCD, *-Chol*), then labelled with Theta*, washed, spread and observed as in Fig. 1. (**a**) Representative imaging of four independent experiments. Y*ellow* and *green arrowheads* indicate respectively LC and HC membrane areas, *scale bars* 2 µm. (**b**) Quantification of membrane average curvature and RBC perimeter of untreated (*open bars*) and treated (*black bars*) healthy and elliptocytotic RBCs. (**c,d**) Relation between lipid domains and RBC deformability. Fresh healthy RBCs were either left untreated *(untreated)* or incubated with mßCD (*-Chol & -SM domains*) or mßCD followed by chol repletion (*-Chol domains*), or maintained at 4 °C during 2 weeks to accelerate aging (*Stored*). All preparations were then labelled with Theta* or BODIPY-SM, washed, spread and observed as in Fig. 1. (**c**) Representative imaging of three independent experiments. Y*ellow* and *green arrowheads* indicate respectively LC and HC membrane areas, *scale bars* 2 µm. (**d**) RBC filtration. The amount of RBCs that passed through the filter was measured by haemoglobin release after complete RBC lysis and expressed as percentage of the amount of RBCs before filtration. Results are means ± SEM of 6–8 filtrations from 2–4 independent experiments. Statistical significance was tested with one-way ANOVA followed by Tukey’s post-hoc test.

The next question was thus to ask whether chol-enriched domain abrogation could affect RBC membrane curvature and deformability. To investigate the importance of chol-enriched domains for membrane curvature changes, healthy RBCs and elliptocytes were treated as previously^12, 14^ with mßCD to deplete membrane chol and abrogate chol-enriched domains, as revealed by the suppression of Theta* labeling (Fig. 5a). This treatment decreased elliptocytotic, but not healthy, membrane curvature rim, without any detectable impact on the RBC perimeter (Fig. 5b).

To evaluate whether chol-enriched domains are involved in RBC deformability, we compared the consequences of combined chol- and SM-enriched domain depletion *vs* specific chol-enriched domain abrogation on RBC deformability tested upon RBC squeezing across tiny pores by filtration. To abrogate both chol- and SM-enriched domains, RBCs were treated with mßCD (Fig. 5c, *-Chol & -SM domains*; Supplementary Fig. 10A and B, central panels). To specifically deplete chol-enriched domains, we used mßCD followed by chol repletion (*mβCD* *→* + *Chol)*. This treatment allows to (i) fully replete membrane chol (Supplementary Fig. 10A, right panel); (ii) fully restore SM-enriched domains, as revealed by both Lysenin* and BODIPY-SM labelling (Fig. 5c, *−Chol domains*; Supplementary Fig. 10Bf,i, yellow arrowheads); and (iii) reform only a small part of chol-enriched domains, *i.e*. those that are localized in the center of the RBC (Supplementary Fig. 10Bc, yellow arrowheads) and co-enriched in chol and SM (data not shown).

Combined chol- and SM-enriched domain abrogation (*mβCD*) and specific chol-enriched domain depletion (*mβCD* → + *Chol*) similarly impaired RBC ability to pass across the filter pores (Fig. 5d). Reduced deformability was similar to that obtained with RBCs maintained for 2 weeks at 4 °C to accelerate aging (*stored at 4 °C*, see below for characterization of RBC modification upon storage), included in the experiment as an internal control for decreased deformability. Altogether, these results indicated that chol-enriched domain abrogation induced the loss of high curvature areas in the rim of elliptocytes and a decreased deformability in healthy RBCs.

### Transient increase of SM-enriched domains and calcium efflux upon RBC shape restoration

**Figure 6:**
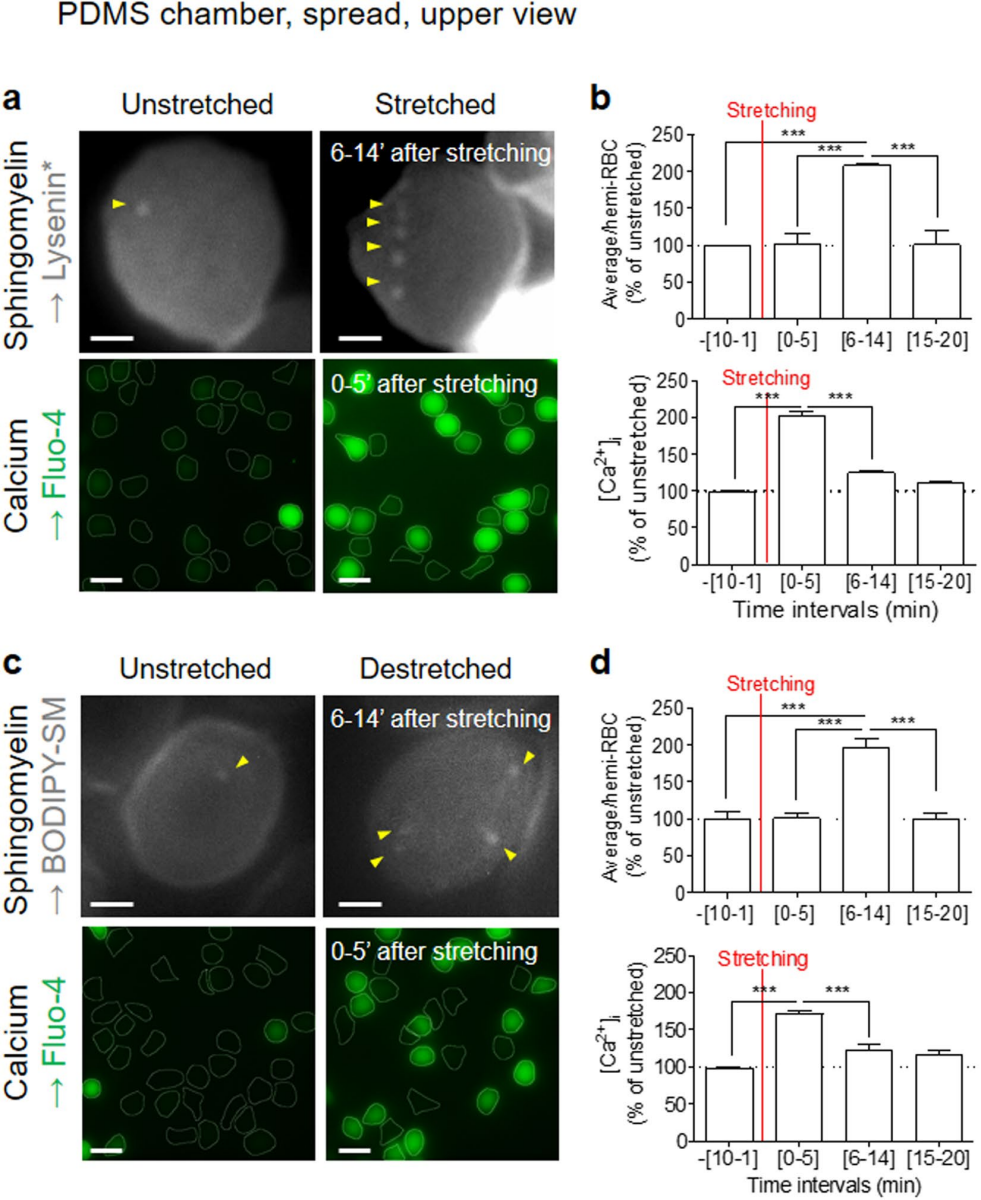
Transient increase of SM-enriched domains and calcium efflux during RBC shape restoration. Fresh healthy RBCs labelled for SM (Lysenin*/BODIPY-SM) or intracellular Ca^2+^ (Fluo-4) and spread onto PLL-coated PDMS chambers were visualized before stretching (*unstretched*) and either after stretching (*stretched*; **a,b**) or upon return to initial shape after a 1-min stretching (*destretched*; **c,d**). (**a,c**) Representative imaging. In *upper panels* (SM labelling), one representative RBC is shown; y*ellow arrowheads* point to domains in LC membrane area. *In lower panels* (Ca^2+^ labelling), several RBCs are shown. Representative imaging of 2–3 independent experiments, s*cale bars* 2 µm in *upper panels* and 10 µm in *lower panels*. (**b**,**d**) Quantification of domain abundance (number by hemi-RBC expressed as percentage of unstretched, *upper panels*) and Fluo-4 intensity (average intensity by hemi-RBC expressed as percentage of unstretched, *lower panels*). Results were grouped by time intervals around stretching time. Results are means ± SEM of 1459–1914 RBCs from 2–3 independent experiments. Statistical significance was tested with one-way ANOVA followed by Tukey’s post-hoc test.

Although SM-enriched domains were not associated with membrane curvature changes, they transiently increased in abundance in the RBC center, starting about 5 min after stretching and lasting around 10 min (Fig. 6a and quantification in Fig. 6b, upper panel). We thus asked whether there could be a relation between SM domain abundance and transient Ca^2+^ exchanges, which are known to be involved in transient volume modulation upon RBC deformation^26^. Ca^2+^ exchanges were determined by RBC labeling with Fluo-4, a fluorescent indicator of intracellular Ca^2+^ concentration ([Ca^2+^]_i_). A [Ca^2+^]_i_ increase was observed in the first 0–5 min after stretching, followed by a decrease during the next 15 min (Fig. 6a and quantification in Fig. 6b, lower panel).

The temporal concordance ([6–14 min]) between increase of SM-enriched domain abundance and stimulation of Ca^2+^ efflux suggested a combined stimulation of these two events upon shape/volume restoration. To assess this hypothesis, BODIPY-SM-labelled RBCs were observed in their resting state (Fig. 6c, *unstretched*), then returned to their initial shape (*destretched*) after a rapid stretching (during 1 min). Destretched RBCs exhibited within a 6–14 min time interval after stretching a simultaneous transient increase of SM-enriched domain abundance and Ca^2+^ efflux (Fig. 6c and quantification in Fig. 6d), showing again the stimulation of SM-enriched domains in shape restoration, along with Ca^2+^ efflux.

### Specific increase of SM-enriched domains upon calcium efflux

**Figure 7:**
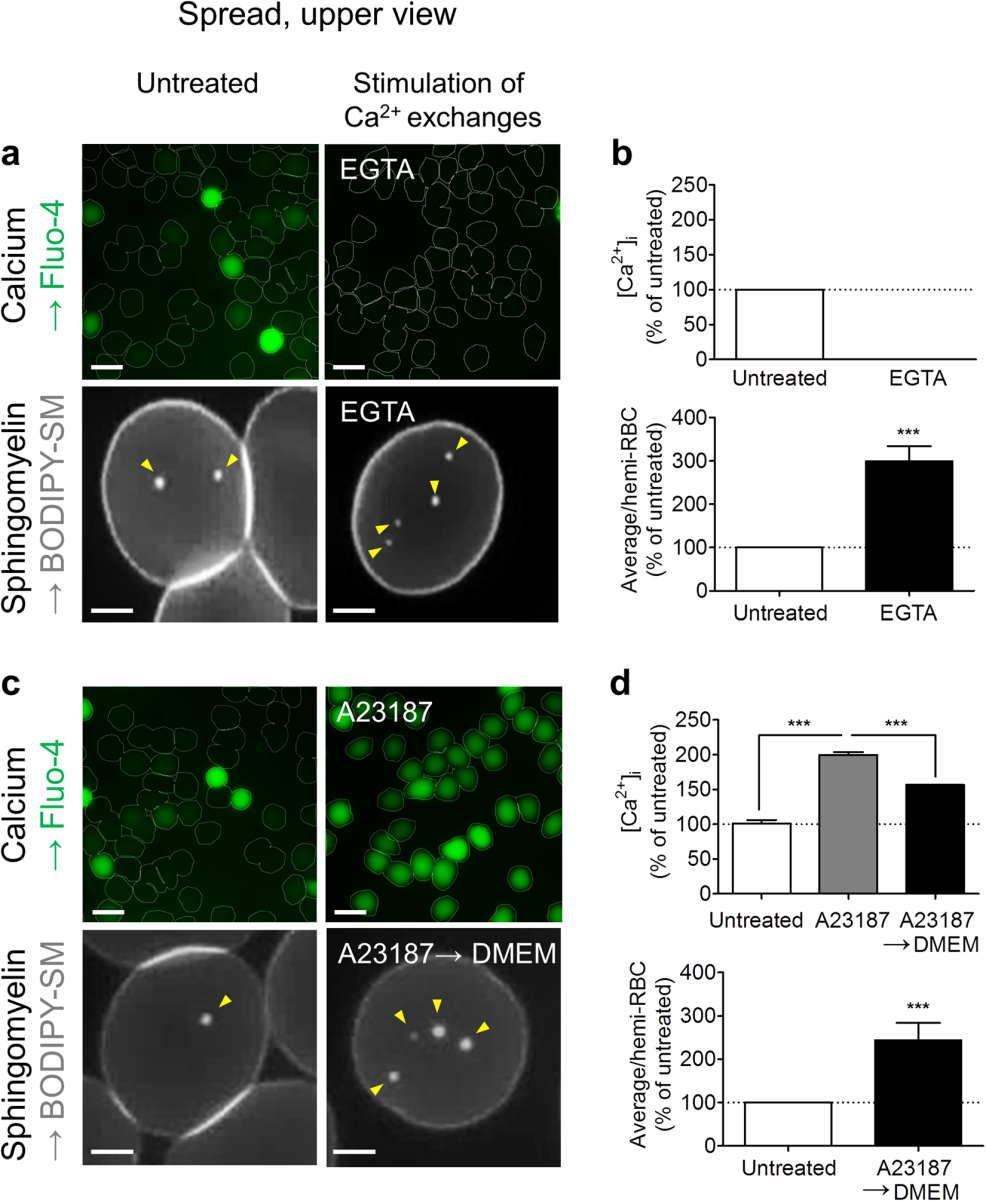
Increase of SM-enriched domains during calcium efflux. Fresh healthy RBCs were either left untreated (*open bars*) or stimulated for Ca^2+^ exchanges (*black & grey bars*), either by incubation with EGTA in Ca^2+^-free medium (**a,b**) or by treatment with the Ca^2+^ ionophore A23187 followed or not by a reincubation in A23187-free medium (**c,d**). RBCs were then labelled with Fluo-4 or BODIPY-SM, washed and either spread onto PLL-coated coverslips and visualized by vital confocal/fluorescence microscopy (**a,c** and *lower panels* in **b,d**) or left in suspension and evaluated for Fluo-4 intensity by spectrophotometry (*upper panels* in **b,d**). (**a**,**c**) Representative imaging. In *lower panels*, on representative RBC; y*ellow arrowheads* indicate domains in LC membrane area. Representative imaging of 3–7 independent experiments, s*cale* 10 µm in *upper panel* and 2 µm in *lower panel*. (**b**,**d**) Quantification of Fluo-4 intensity (*upper panels*; average intensity by sample expressed as a percentage of untreated RBCs) and domain number (*lower panels*; average number by hemi-RBC expressed as a percentage of untreated RBCs). Results are means ± SEM of 11–12 samples (*upper panels*) and 82–1654 RBCs (*lower panels*) from 3–7 independent experiments. Statistical significance was tested with two-sample t-tests, except one-way ANOVA followed by Tukey’s post-hoc test in *upper panel* in d.

We next investigated if SM-enriched domain stimulation and secondary Ca^2+^ efflux, observed during shape restoration, could be related. Ca^2+^ efflux was induced by RBC incubation in Ca^2+^-free medium supplemented with EGTA, a Ca^2+^-chelating agent (Fig. 7a and b, upper panels). An increased abundance of SM-enriched domains was observed in the RBC concavity (Fig. 7a and b, lower panels). This effect was reversible (data not shown), excluding toxicity. In contrast, the abundance of chol-enriched domains was not significantly increased (Supplementary Fig. 11).

To better mimic Ca^2+^ exchanges occurring during RBC deformation, RBCs were incubated with a very low concentration of the Ca^2+^ ionophore A23187 to induce [Ca^2+^]_i_ increase (*A23187*) without hemolysis (data not shown), then in A23187-free medium (*A23187 → DMEM*) to favor secondary Ca^2+^ efflux (Fig. 7c and d, upper panels, quantifications after 10 min in A23187-free medium). This experiment confirmed the increased abundance of SM-enriched domains in the RBC concavity upon Ca^2+^ secondary efflux (Fig. 7c and d, lower panels). Altogether, our results indicated that SM-, but not chol-, enriched domains were stimulated during Ca^2+^ efflux.

### Impairment of intracellular calcium concentration and volume increase ability upon SM-enriched domain abrogation

**Figure 8:**
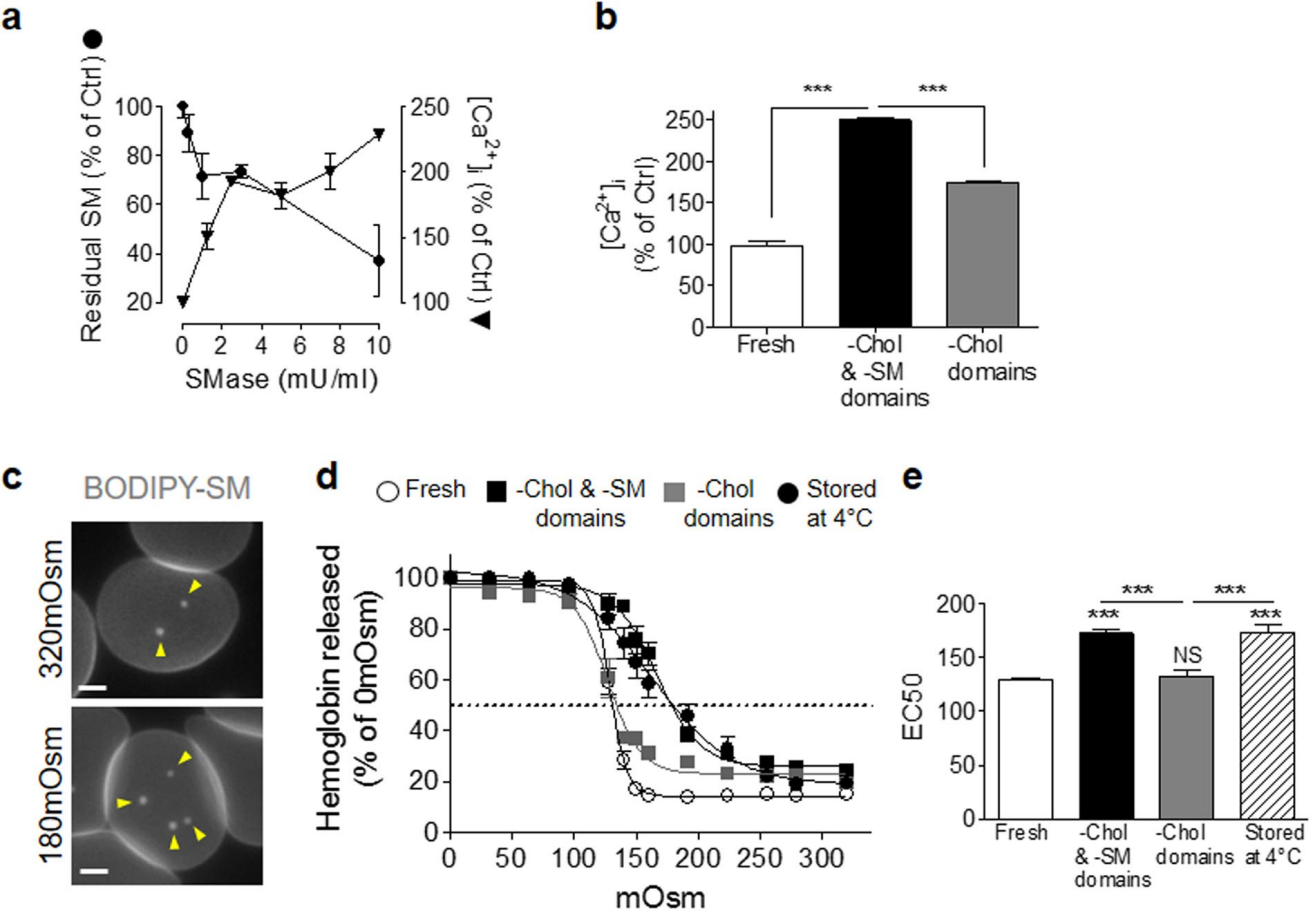
Impairment of intracellular calcium concentration and volume increase ability upon SM-enriched domain abrogation. (**a**) Fresh healthy RBCs were preincubated with Fluo-4, treated with the indicated concentrations of sphingomyelinase *(SMase)* to deplete endogenous SM and evaluated for SM residual content (*black circles*) and [Ca^2+^]_i_ (*black triangles*). Results are expressed as percentage of control *(0 mU/ml SMase)* and are means ± SEM from three independent experiments. (**b**) RBCs were preincubated with Fluo-4, treated to abrogate both chol and SM domains (*-Chol & -SM domains*) or chol domains only (*-Chol domains*) as described in Fig. 5 and then evaluated for [Ca^2+^]_i_. Results are expressed as percentage of untreated RBCs and are means ± SEM of 8 samples from two independent experiments. (**c**) RBCs were labelled with BODIPY-SM, washed, spread onto PLL-coated coverslips and visualized by fluorescence microscopy in media of indicated osmolarity. Representative imaging of 4 independent experiments, *scale bars* 2 µm. *Yellow arrowheads* point to the central area of the RBC membrane. (**d,e**) Fresh RBCs treated for domain abrogation (as in Fig. 5c) or stored at 4 °C were incubated in media of indicated osmolarity and evaluated for haemoglobin release (d, average haemoglobin absorbance by sample expressed as a percentage of 0 mOsm; e, EC50). Results are means ± SEM of 12–18 samples from 4–6 independent experiments. Statistical significance was tested with one-way ANOVA followed by Tukey’s post-hoc test.

We finally asked whether Ca^2+^ efflux and volume increase, two processes occurring during shape restoration, could be impaired upon lipid domain abrogation^26, 27^. We therefore measured [Ca^2+^]_i_ in RBCs treated with increasing concentrations of sphingomyelinase, which converts SM into ceramide. Between 1 and 10 mU/ml, this treatment decreased the SM level from ~10 to 60% and induced a concomitant increase of [Ca^2+^]_i_ (Fig. 8a), indicating the inverse relationship between SM membrane level and [Ca^2+^]_i_. Because sphingomyelinase is able to abrogate both SM- and chol-enriched domains^12, 14^, we then evaluated [Ca^2+^]_i_ upon both chol- and SM-enriched domain abrogation (*-Chol & -SM domains*) and upon specific chol-enriched domain depletion (*-Chol domains*). Abrogation of both chol- and SM-enriched domains induced an increase of [Ca^2+^]_i_, which was significantly restored 10 min after SM-enriched domain repletion (Fig. 8b, *-Chol domains*).

Since RBC volume increase seems to be at the center of the relation between SM-enriched domains and Ca^2+^ efflux during RBC shape restoration, we then evaluated lipid domain abundance and hemoglobin release upon RBC incubation in media of decreasing osmolarity, a simple way to evaluate RBC ability to increase their volume^30^. RBCs incubated in iso- to hypo-osmotic media (320 mOsm to 180 mOsm) increased SM-enriched domain abundance in the concavity of the cell (Fig. 8c), as a first line of evidence for the relation between volume increase and SM-enriched domain stimulation. We then evaluated the impact of chol- and SM-enriched domain abrogation (*-Chol & -SM domains*) and specific chol-enriched domain depletion (*-Chol domains*) on hemoglobin release by RBCs incubated in media of decreased osmolarity. As compared to untreated fresh RBCs, chol- and SM-enriched domain abrogation (*-Chol & -SM domains*) and RBC storage at 4 °C, but not chol-enriched domain depletion only (*-Chol domains*), impaired resistance to hemolysis (Fig. 8d,e). Altogether these experiments confirmed the relation between SM-, but not chol-, enriched domains and the regulation of [Ca^2+^]_i_ and volume increase.

### Specific vesiculation of chol- and SM-enriched domains upon aging

**Figure 9:**
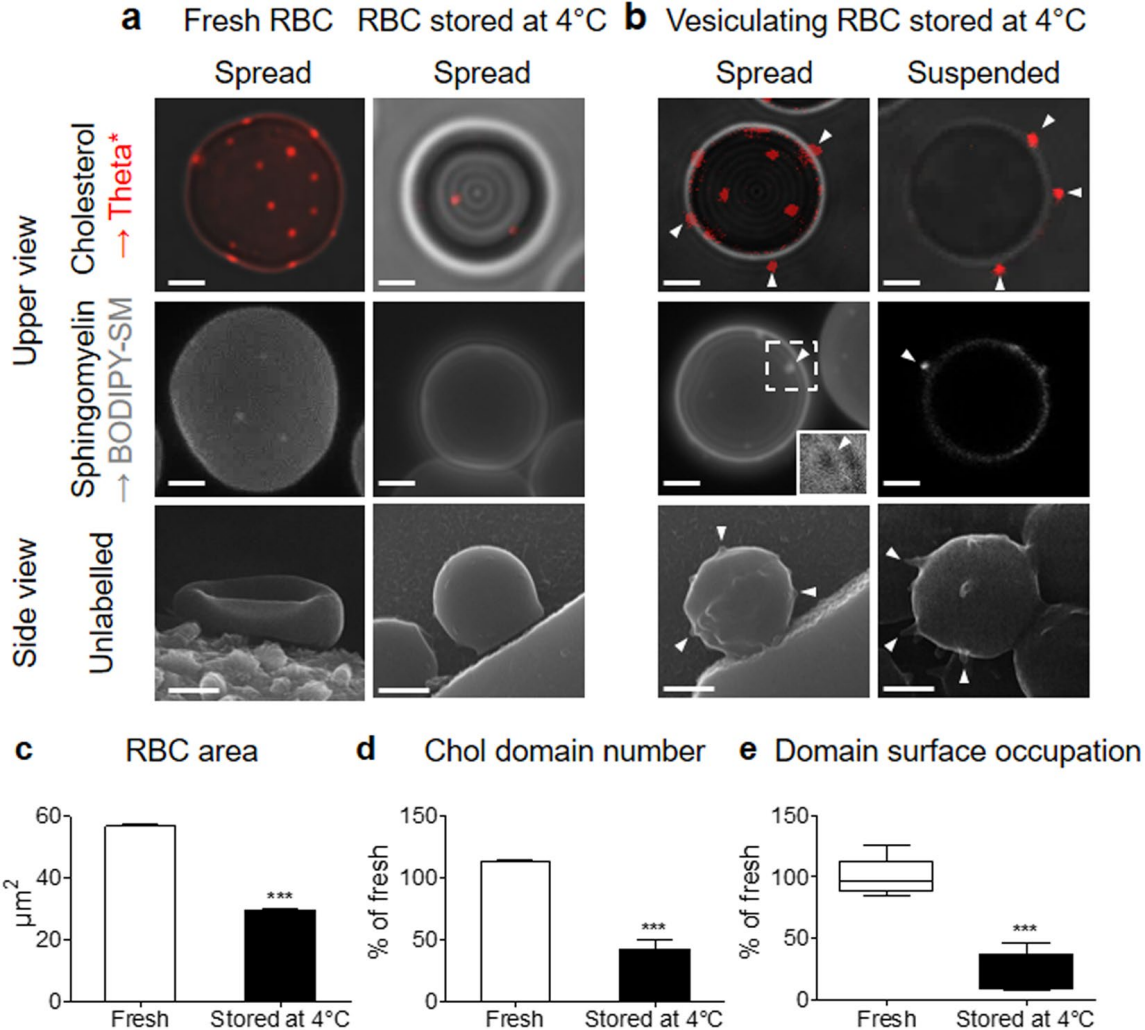
Specific vesiculation of chol- and SM-enriched domains upon storage at 4 °C. Fresh and stored (15 days at 4 °C) healthy RBCs were labelled (**a** and **b**, *upper and central panels*) or not (**a** and **b**, *lower panels*), left in suspension *(suspended)* or spread (*spread*) and observed by vital confocal/fluorescence microscopy (**a** and **b**, *upper and central panels*) or SEM (**a** and **b**, *lower panels*). (**a**,**b**) Representative imaging. *White arrowheads* point to membrane vesiculation (**b**). SM-enriched vesiculating domain in spread RBC is boxed and can be observed by specific transmission pattern (see 2x zoom in right lower corner of the image). Representative images of 2–3 independent experiments, *scale bars* 2 µm. (**c**–**e**) Quantification of RBC area (c; µm^2^ by hemi-RBC), chol-enriched domain number (d; average by hemi-RBC expressed as percentage of fresh RBCs) and domain surface occupation (e; total domain area by hemi-RBC area, expressed as percentage of fresh RBCs). Results are means ± SEM of 128–252 RBCs from two independent experiments. Statistical significance was tested with two-sample t-tests.

Upon senescence *in vivo*, RBCs undergo multiple changes including the loss of membrane by vesiculation and alterations in cell volume, density and deformability^31, 32^. Different experimental conditions have been developed in the literature to investigate the mechanisms underlying the RBC vesiculation process, including blood storage at 4 °C. Several lines of evidence indicate that the changes to the RBC membrane during blood storage somehow mimick *in vivo* RBC senescence mechanisms^27, 33, 34^. Accordingly, after 15 days of RBC storage at 4 °C, we observed: (i) an increased susceptibility to vesiculation, as shown by scanning electron microscopy on unlabeled RBCs (Fig. 9b, lower panels & Supplementary Fig. 12Ab); (ii) a decreased membrane area (Fig. 9a, *upper and central panels*; quantification in c) and biconcavity (Fig. 9a, *lower panels*) together with an increase of RBC circularity (Supplementary Fig. 12B); (iii) a decreased deformability, as revealed by filtration through polycarbonate filters (Fig. 5); (iv) phosphatidylserine (PS) exposure to the outer PM leaflet, as revealed by Annexin V labeling (Supplementary Fig. 12Ad); (v) Band 3 aggregates, thanks to maleimide labelling (Supplementary Fig. 12Af); and (vi) an increased intracellular Ca^2+^ concentration (Supplementary Fig. 12C).

We then analyzed lipid domains in RBCs stored at 4 °C to assess if they could be involved in membrane vesiculation upon aging. Stored RBCs lost both types of lipid domains (Fig. 9a, *upper and central panels*; quantification for chol-enriched domains in d). We believe that such loss was not due to Toxin* labelling, as vesiculation was also observed on unlabelled RBCs (Fig. 9b, *white arrowheads at lower panels*), but resulted from specific domain vesiculation (arrowheads at Fig. 9b, *white arrowheads at upper and lower panels*). Of note, the significant decrease of total domain occupation of the RBC surface in stored *vs* fresh RBCs suggested a higher sensitivity to vesiculation of domains *vs* surrounding membrane (bulk) (Fig. 9e).

## Discussion

Although PM lipid domains have been evidenced in several living cells, their functions remain largely unclear. We here investigated whether they could contribute to function-related cell (re)shaping processes, a central role of the PM. To assess this question, we used erythrocytes since they (i) exhibit a specific biconcave shape; (ii) expose drastic reshaping upon both reversible deformation and membrane vesiculation upon aging; (iii) display at their outer PM submicrometric domains differentially enriched in chol and SM; and (iv) offer a featureless surface and no lipid trafficking which facilitate the study of these domains.

Deciphering the importance of lipid domains for cell (re)shaping requires specific, non-toxic, sensitive and quantitative tools compatible with live cell imaging. Hence, these probes should decorate and not induce domains. Since the small fluorescent SM analog (BODIPY-SM) and the large Toxin Lysenin* detect spatially undistinguishable domains at the RBC surface whatever the order of labelling, we suggested that mCherry-Lysenin﻿ Toxin fragment does not trigger but rather reveals preexisting submicrometric domains^12^. RBC labeling with low-molecular weight fluorescent chol reporters, such as the polyene antibiotic filipin and dehydroergosterol, did not produce significant signal without obvious signs of RBC abnormal shape (our unpublished data), precluding comparison with Theta* for analysis of chol lateral heterogeneity. We however observed that sphingomyelinase treatment has no effect on total Theta* binding but fully abrogates submicrometric domains^14^, implying that chol-enriched domains critically depend on endogenous SM and further suggesting that, at least upon SM depletion, Theta* by itself is unable to trigger domain formation. We also excluded the possibility that SM-enriched domains track cell curvature due to Lysenin* binding. Indeed, the specific association of SM-enriched domains with RBC low curvature areas was similarly observed with the small probe BODIPY-SM. We were not able to test this possibility for chol-enriched domains due to the reasons mentioned above.

**Figure 10:**
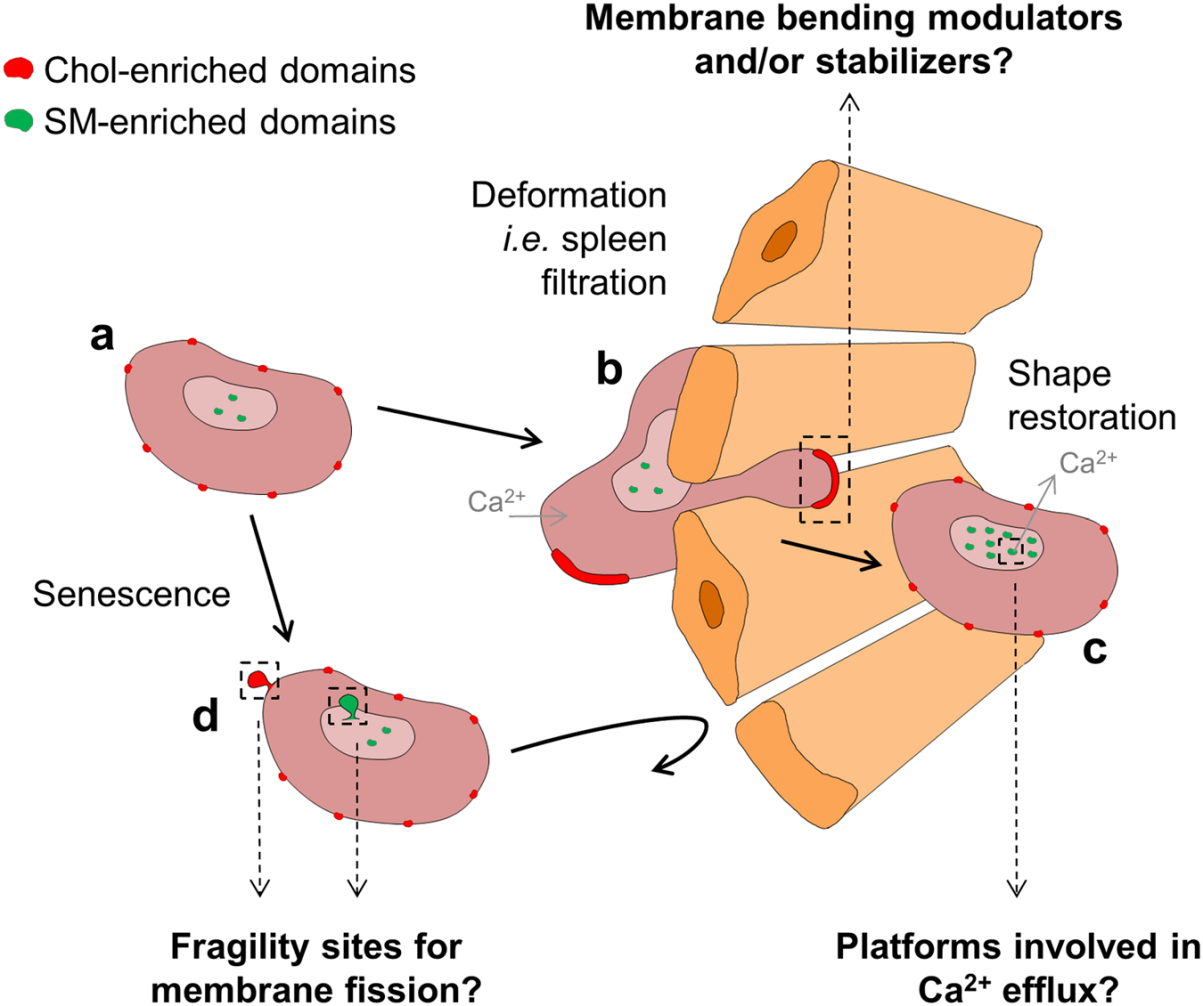
Model summarizing the relation between chol- and SM-enriched submicrometric domains and RBC (re)shaping and their hypothetical implication in these processes (dashed arrows). (**a**) In fresh healthy RBCs, chol- (*red*) and SM- (*green*) enriched submicrometric domains associate to distinct membrane areas with high and low curvature, respectively. (**b,c**) Upon deformation, chol-enriched domains are recruited to the edges and gather in increased curvature areas of the membrane. During shape and volume restoration after deformation, SM-enriched domains increase in abundance in the concavity of the RBC, along with Ca^2+^ efflux. (**d**) During RBC aging, both lipid domains appear as specific sites for membrane loss by vesiculation. From our data we suggest that PM lipid domains could contribute to RBC reshaping as membrane bending modulator/stabilizer upon deformation, platforms allowing for Ca^2+^ efflux during the following volume and shape restoration and fragility sites for membrane vesiculation upon aging but these hypotheses need further investigation.

Using these two specific Toxin* fragments and/or BODIPY-SM, we first highlighted the preferential association of chol- and SM-enriched submicrometric domains with high and low membrane curvature areas of the RBC biconcave membrane, respectively (Fig. 10a). RBC biconcavity was not impacted by domain abrogation, suggesting that chol- and SM-enriched domains are not actively involved in RBC specific biconcave membrane shape. Instead, the specific association of lipid domains to distinct membrane curvature areas could suggest that lipid domain topography is controlled by the RBC membrane curvature. While no data are currently available on eukaryotic living cells, experiments on model membranes made of lipid mixtures indicate that a curvature-driven lipid domain sorting is possible, on model membranes with preexisting phase separation or not^35–37^. Mechanistically, lipid domains seem not effectively curvature-sorted according to the individual lipid shape, but instead by cooperative properties of lipid domains, such as domain intrinsic curvature or order and correlated bending stiffness^35–37^. Taking into account the high order of chol-enriched domains in model membranes, their association with high curvature areas is surprising. However, unfavorable properties like bending rigidity could be circumvented by domain-associated binding proteins that are curvature sorted^38^ or the line tension at domain boundary^39^. It should be nevertheless stressed that the absolute values of the RBC PM curvatures are very small as compared to domain size, when comparing with the mentioned experiments on model membranes (tubes pulled from GUVs or surfaces imposing curvature to lipid bilayers), questioning the possibility of such curvature-driven mechanism. Non-mutually exclusive additional mechanisms could be proposed, such as the cytoskeleton and transmembrane protein pinning^38^ or interleaflet coupling.

After investigating the relation between PM lipid domains and RBC specific shape, we turned to analyze their potential implication in RBC reshaping during (i) deformation, (ii) shape restoration after deformation, and (iii) local membrane vesiculation upon aging. First, we observed the recruitment of chol-enriched domains in areas of increased membrane curvature created in the rim of elliptocytes and of healthy RBCs stretched on PDMS chambers (Fig. 10b). Hence, specific abrogation of chol-enriched domains both decreased RBC deformability and abrogated elliptocyte high curvature areas, suggesting that chol-enriched domains could contribute to RBC deformability as membrane bending modulator/stabilizer. Indeed, microfluidic deformation of GUVs shows the gathering and reorganization of lipid phases, which could be favorable due to the reduction of the line tension at domain boundary^40^. However, RBCs are by far more complex than GUVs due to the presence of membrane proteins and cytoskeleton, which are known to be involved in membrane bending and RBC deformation. The potential contribution of those actors remains to be determined, in order to decipher whether lipid domains are primary actors in membrane deformation through the modulation/stabilization of membrane curvature or not.

Second, we highlighted the increase of SM-enriched domains in RBC concavity along with secondary Ca^2+^ efflux during shape restoration after deformation. It is tempting to propose that SM-enriched domains are involved in secondary Ca^2+^ efflux allowing for shape and volume restoration after deformation (Fig. 10c), based on the following lines of evidence: (i) the same specific increase of SM-enriched domain abundance was observed upon Ca^2+^ efflux stimulation with either A23187 or EGTA treatments, (ii) [Ca^2+^]_i_ increase was observed upon SM- and chol-enriched domain abrogation, which was partially restored after specific SM-enriched domain repletion, (iii) RBC ability for volume increase in hypo-osmotic medium was impaired upon combined chol- and SM-enriched domain abrogation, but was recovered upon specific SM-enriched domain repletion, and (iv) upon RBC membrane mechanical deformation, a transient [Ca^2+^]_i_ increase is observed, followed by the secondary activation of the Gardos channel leading to a transient volume decrease^26^. In this context, SM-enriched domains could potentially represent platforms for the sorting and/or activation of Ca^2+^ efflux membrane proteins.

Third, we revealed the specific loss of chol- and SM-enriched domains upon storage at 4 °C (mimicking aging), suggesting that chol- and SM-enriched domains could represent sites for vesiculation upon aging and storage (Fig. 10d). Accordingly, chol-enriched domains have been proposed to be the sites for membrane fusion upon HIV infection^41^. Theoretical works^42^ and biophysical experiments on model membranes^39, 41^ propose the line tension associated to domain boundary is responsible for specific lipid domain vesiculation. In this context lipid domains could be specific fragility sites for membrane vesiculation due to line tension at domain boundary.

To the best of our knowledge, this is the first comprehensive study reporting on living eukaryotic cells the implication of different lipid domains in cell reshaping processes (Fig. 10a–d). From our data it is tempting to propose that PM lipid domains contribute to RBC reshaping as membrane bending modulator/stabilizer upon deformation, platforms allowing for Ca^2+^ efflux during volume and shape restoration and fragility sites for membrane vesiculation upon aging (Fig. 10a–d). However, several unanswered questions remain: (i) are the domains primary actors or not; (ii) to what extent do these domains act on, or are recruited by, membrane proteins or the cytoskeleton; and (iii) whether these mechanisms are relevant to conditions closer to RBC physiological environment (*e.g*. at 37 °C and under application of a shear stress). While unveiling their functions is the first step for understanding lipid domains, it raises the issue of underlying mechanisms of action and energy considerations/driving forces. We hope our model, by providing two types of optically resolved lipid domains with distinct composition, topography and potential functions, could help progressing towards these questions.

## Methods

### Red blood cell isolation

This study was approved by the Medical Ethics Institutional Committee of the Université catholique de Louvain; each donor gave written informed consent. All methods were performed in accordance with the relevant guidelines and regulations. RBCs were freshly isolated from healthy volunteers and one patient with hereditary elliptocytosis. Blood was collected by venopuncture into dry K^+^/EDTA-coated tubes. For each experiment, blood was diluted 1:10 in Dulbecco’s Modified Eagle Medium (DMEM containing 25 mM glucose, 25 mM HEPES and no phenol red, Invitrogen), then washed twice by centrifugation at 133 *g* for 2 min and resuspension. Washed RBCs were kept at 2 * 10^7^ cells/ml (washed RBCs:medium ratio of 1:11, *v:v*), then incubated or not with pharmacological agents and measured for Ca^2+^ or hemoglobin release or imaged by confocal/fluorescence or scanning electron microscopy (see below). Most experiments were carried out on fresh RBCs, except for the aging experiment. For the later study, healthy RBCs were maintained during 2 weeks at 4 °C into the K^+^/EDTA-coated tubes.

### Calcium modulation and measurement

Modulation of the [Ca^2+^]_i_ was performed on washed RBCs in suspension at room temperature (RT) under continuous agitation in (i) DMEM containing 0.3 µM of the Ca^2+^ ionophore A23187 (Sigma-Aldrich) for 10 min; (ii) A23187-containing DMEM for 10 min followed by reincubation in A23187-free medium for another 10 min; or (iii) Ca^2+^-free homemade medium containing 1 mM Ca^2+^-chelating agent ethylene glycol-bis(β-aminoethyl ether)-N,N,N’,N’-tetraacetic acid (EGTA, Sigma-Aldrich) for 10 min. All RBCs were then pelleted at 133 *g* for 2 min, resuspended in the adequate medium and either used in suspension for [Ca^2+^]_i_ measurement or hemoglobin release or labelled, spread onto PLL-coated coverslips and observed by vital microscopy for lipid domains (see below) or Ca^2+^ determination. To evaluate [Ca^2+^]_i_, washed RBCs were preincubated in suspension at RT with 5 µM Fluo-4 (Invitrogen) in Ca^2+^-free medium for 60 min under continuous agitation, pelleted at 133 *g* for 2 min and resuspended in Ca^2+^-free medium, then treated with the above modulators and either measured for Ca^2+^ in 96-well plates (excitation 490 nm, emission 520 nm; SpectraCountTM, Packard BioScience Co.) or spread onto PLL-coated coverslips for intracellular Ca^2+^ signal (see below).

### SM and chol content modulation and measurement

To modulate SM or chol contents, washed RBCs were preincubated in suspension at RT in DMEM containing 1 mg/ml bovine serum albumin (BSA; Sigma-Aldrich) supplemented with (i) 0–10 mU/ml Bacillus cereus sphingomyelinase (Sigma-Aldrich) for 10 min; (ii) 0.5 mM or 0.9 mM methyl-ß-cyclodextrin (mβCD; Sigma-Aldrich) for 30 min; or (iii) 0.9 mM mβCD, followed by repletion with 3.5 µg/ml mβCD:chol (Sigma-Aldrich) for 60 min. All RBCs were then pelleted at 133 g for 2 min, resuspended in DMEM and either used in suspension for hemoglobin release (see below) or Ca^2+^ (see above) or spread onto PLL-coated coverslips for vital imaging of lipid domains (see below). SM and chol contents were determined as previously described^14, 43^.

### RBC labelling and vital fluorescence/confocal imaging

Washed RBCs were labeled with Toxin* fragments or BODIPY-SM. Lysenin* and Theta* were produced as previously described^12, 14^, dissolved in 1 mg/ml DMEM-BSA and cleared of aggregates before each experiment by centrifugation at 20,000 *g* for 10 min. RBC labeling with Toxins* was performed in suspension (*i.e*. before immobilization) with either 1.25 µM Lysenin* or 0.55 µM Theta* in DMEM/BSA at 20 °C for 25 min under continuous agitation, then pelleted at 133 *g* for 2 min and resuspended in DMEM. RBC labeling with BODIPY-SM (Invitrogen) was performed after RBC immobilization on coverslips at 0.75 µM at 20 °C for 15 min. To immobilize RBCs for imaging we developed two complementary systems, RBC spreading onto poly-L-lysine (PLL, 70–150 kDa; Sigma-Aldrich)-coated coverslips and RBCs in suspension. For spread RBCs, coverslips were first coated with PLL:DMEM (1:1, *v:v*) at 37 °C for 40 min, then washed with DMEM at 20 °C for 5 min. Labelled RBCs were then dropped into the coated coverslips at 20 °C for exactly 4 min, the suspension was removed and replaced by fresh medium, and attached RBCs were allowed to spread for another 4 min. The coverslip was placed upside down on a Lab-Tek chamber and then observed. For the “in suspension” system, labelled RBCs were dropped to settle down in µ-Slide VI^0.4^ uncoated IBIDI chambers (IBIDI, Proxylab; 100 µl by channel). All preparations were examined at RT either with a Zeiss LSM510 confocal microscope using a plan-Apochromat 63X NA 1.4 oil immersion objective or with a Zeiss wide-field fluorescence microscope (Observer.Z1) using a plan-Apochromat 100X/1.4 oil Ph3 objective.

### RBC (de)stretching on PDMS chambers

Deformation experiments were conducted by spreading Toxin*/BODIPY-SM- or Fluo-4-labelled RBCs on a 4 cm^2^ PLL-coated polydimethylsiloxane (PDMS) stretchable chamber (B-Bridge, Gentaur). Washed RBC labelling with Toxin* or Fluo-4 was done in suspension before spreading whereas labelling with BODIPY-SM was performed after spreading on the PDMS chamber. Briefly, PDMS chamber was first coated with PLL:DMEM (1:1, *v:v*) at 37 °C for 40 min, washed with DMEM at 20 °C for 5 min and fixed to the stretching device (STREX, cell strain instrument, B-Bridge). Labelled RBCs were plated into the PDMS chamber for exactly 5 min, then the suspension was removed and replaced by fresh medium, and attached RBCs were allowed to spread for another 5 min. The PDMS chamber was then immediately observed at RT without stretching (unstretched) with a Zeiss wide-field fluorescence microscope (Observer.Z1) using a plan-Neofluar 63X/0.75 Ph2 objective. Stretching and destretching of the chamber were thereafter respectively performed by (i) axial stretching of the right side of the PDMS chamber from 7–17% of the chamber length (stretching); and (ii) return to the initial state after a quick (1 min) axial stretching of the right side of the PDMS chamber from 7–17% of the chamber length (destretching).

### RBC separation by filtration

3 ml of washed RBCs, then 2 ml of DMEM for washing, were forced to pass through a polycarbonate filter with 1.2 µm-pores (ipPORE) by applying a pressure below 0.2 bar. RBCs that passed through the filter were quantified by achieving full hemolysis with 0.2% Triton X-100. The quantity of recovered RBCs was expressed as percentage of total RBCs before filtration.

### Measurement of hemoglobin release

Measurement of hemoglobin released in supernatants was used for evaluating innocuity of pharmacological treatments to RBCs and RBC resistance to hemolysis in media of decreased osmolarity. The latter was determined by incubation of washed RBCs in media of decreasing osmolality (from 320 to 0 mOsm, by dilution of DMEM with demineralized water) for 10 min at RT. Hemoglobin released in supernatants was then read at 560 nm in 96-well plates (SpectraCountTM, Packard BioScience Co.). For normalization, full hemolysis was achieved by 0.2% Triton X-100.

### Scanning electron microscopy

Washed RBCs were spread onto PLL-coated nude grids and quickly washed three times with DMEM. Cells were then fixed by 1% glutaraldehyde (Fluka-Sigma) and post-fixed with 1% (w/v) osmium tetroxide in 0.1 M cacodylate buffer. Samples were washed three times in 0.1 M cacodylate buffer, then in water, dryed overnight and dehydrated in graded ethanol series and critical-point dried (Leica EM CPD030, Vienna, Austria). A 10-nm gold film was sputter-coated (Leica EM MED020, Vienna, Austria) and specimens were observed in a CM12 electron microscope (Philips, Eindhoven, Netherlands) at 80 kV with the use of the secondary electron detector as described^15^.

### Image analysis

Measurements of (i) RBC total projected area (referred to as hemi-RBC), circularity and membrane curvature; (ii) abundance and size of chol- and SM-enriched domains; and (iii) distribution of domains at the lateral PM were performed on high-resolution confocal or epifluorescence images. Except for domain abundance which was assessed by manual counting, all other analyses were done on ImageJ software. Briefly, domains and RBC projected contour were either detected on fluorescent channel images (or transmission images for RBC projected contour when no continuous labeling of the RBC membrane was observed), using analyzed particles plugging, or manually drawn if no detection was possible. These selections were then used for all the following analyses. Prior to contour selection, threshold of both fluorescent and transmission images were adjusted using default methods in ImageJ and were converted in binary images. Domain area, total RBC projected area and RBC membrane circularity were measured with the analyzed particles plugging. Domain occupation of RBC surface was calculated by expressing total domain area of each RBC as a percentage of the corresponding RBC area. Distribution of chol domains at the lateral PM was performed on untresholded fluorescent channel images and that of SM domains on untresholded fluorescent channel images after applying a mask allowing to keep only the domain intensity, both using ImageJ plot profile plugging. Membrane curvature was analyzed using the last version of the Shape Analysis by Fourier Descriptors computation plugging for ImageJ developed by Thomas Boudier. The average curvature associated to chol- and SM-enriched domains and surrounding bulk was obtained by combining results from intensity profiles and curvature profiles.

### Statistical analysis

Values are presented as means ± SEM. Statistical significance was tested either with two-sample t-test or one-way ANOVA followed by Tukey’s post-hoc test (NS, not significant; *p < 0.05; **p < 0.01 and ***p < 0.001).

## Electronic supplementary material

Supplementary figures
