# Supplementary material for: Contribution of plasma membrane lipid domains to red blood cell (re)shaping
Source: Sci Rep. 2017 Jun 27;7:4264. doi: 10.1038/s41598-017-04388-z (PMC5487352; doi:10.1038/s41598-017-04388-z)
Supplement: Supplementary file 1 — Supplementary figures [file 41598_2017_4388_MOESM1_ESM.pdf]

Contribution of plasma membrane lipid domains to red blood cell (re)shaping

Leonard C, Conrard L, Guthmann M, Pollet H, Carquin M, Vermylen C, Gailly P, Van Der Smissen P, Mingeot-Leclercq MP, Tyteca D

Supplementary figures

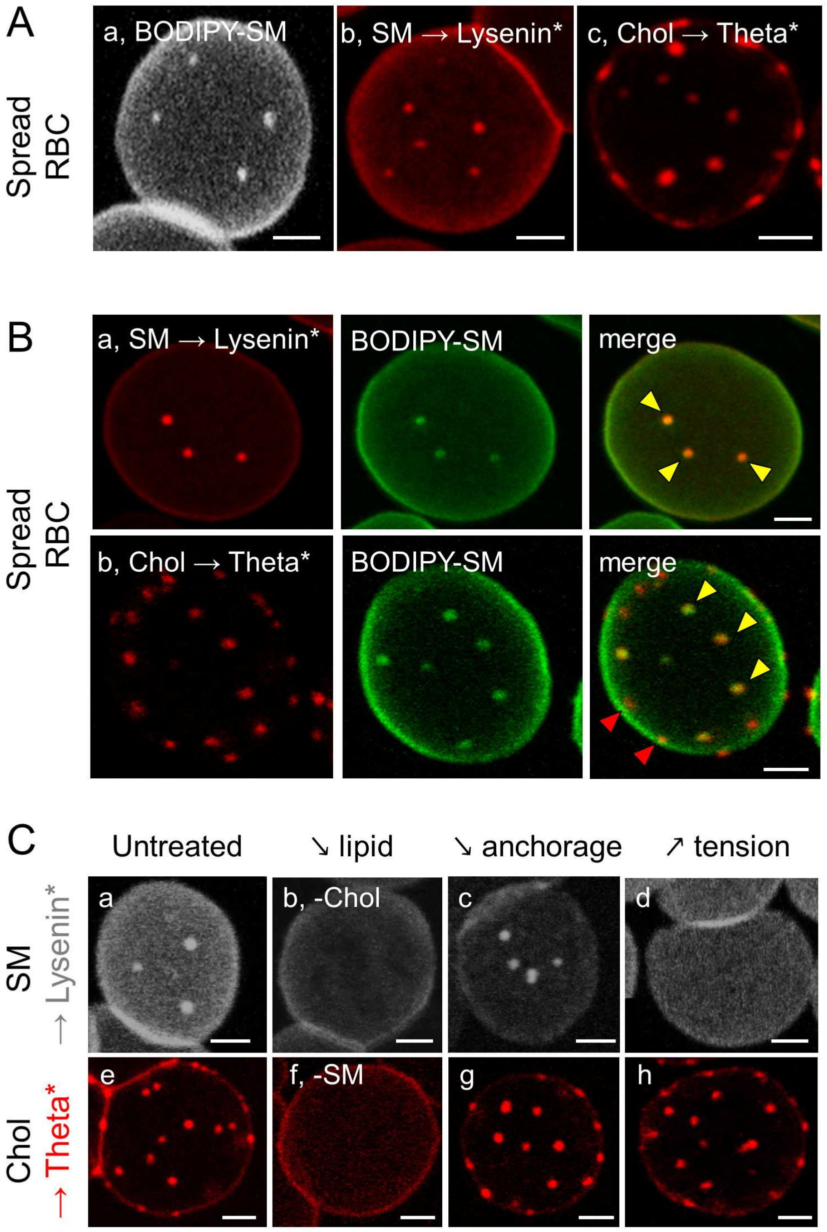

Figure S1. Evidence, spatial relation and regulation of SM- and chol-enriched submicrometric domains at the living RBC plasma membrane. (A) Evidence on spread RBCs. Trace PM insertion of BODIPY-SM (a) or labeling by fluorescent Toxin fragments of endogenous SM (Lysenin*; b) and chol (Theta*; c). RBCs were spread and then analysed by confocal vital imaging. **(B)** Spatial relation. RBCs were labelled by Lysenin* (a; endogenous SM) or Theta* (b; endogenous chol), then by exogenous BODIPY-SM. Whereas Lysenin* and BODIPY-SM perfectly co-localize (a), two types of chol-enriched domains, enriched in either both chol and SM (yellow arrowheads, b) or chol mainly (red arrowheads, b), coexist. **(C)** Regulation. RBCs were either left untreated (a,e; visualization at 20°C) or modulated for: (b,f) endogenous chol or SM content (c,g), membrane:cytoskeleton anchorage at 4.1R complexes (PKC activation), or (d,h) membrane tension (increased spreading on PLL-coated coverslips). All preparations

were then labelled with either Lysenin* (a-d) or Theta* (e-h), spread and visualized as at A. Notice similar lipid domain abrogation upon specific lipid depletion (b,f) and similar lipid domain increase upon acute uncoupling of membrane:cytoskeleton anchorage (c,g) but differential lipid domain modulation by increased tension (d,h). *All scale bars* 2µm. Adapted from [12,15].

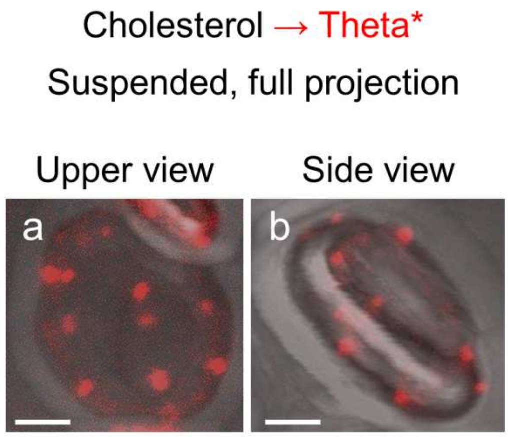

Figure S2 (extension of Fig. 1). Full projection of Theta*-labelled suspended RBCs, observed in upper or side view, reveals similar abundance of chol-enriched domains than spread RBCs. Fresh healthy RBCs were labelled with Theta*, washed, left in suspension in IBIDI chambers and directly visualized in their upper (**a**) or side view (**b**) by vital confocal microscopy. Z-stack images were reconstructed using ImageJ software. S*cale bars* 2 µm.

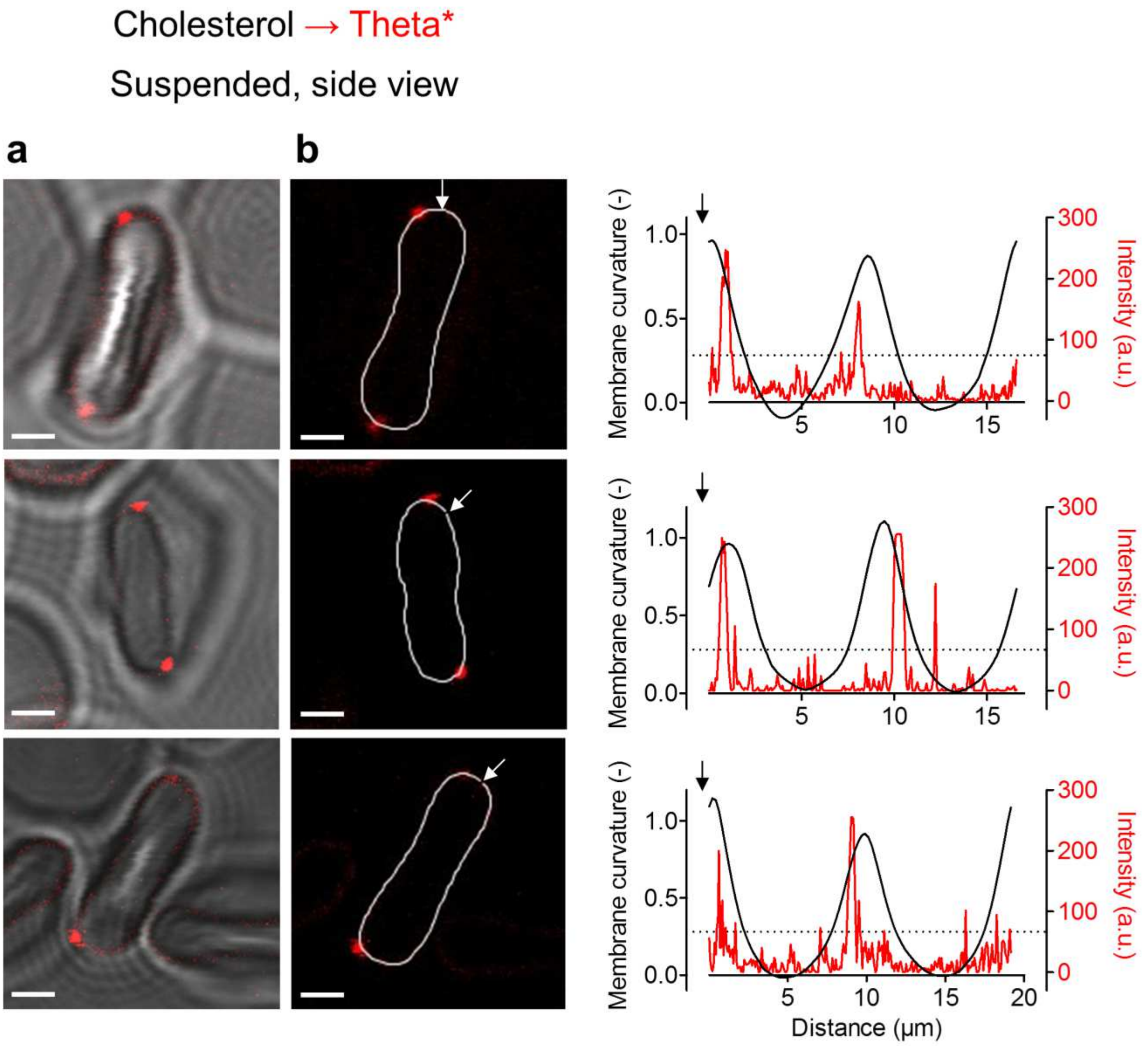

Figure S3 (extension of Fig. 2). Specific association of chol-enriched domains with high curvature areas of the RBC biconcave membrane: gallery of images and profiles. Fresh healthy RBCs were labelled with Theta*, then visualized in suspension by vital confocal microscopy. (**a**) Representative imaging, s*cale bars* 2 µm. (**b**) Profiles comparing Theta* intensity (*red*; *right panels*) and membrane curvature (*black)*. Profiles along paths indicated in *left panels* by *white curved lines* on side-viewed suspended RBCs (counterclockwise from starting point indicated by *white arrows*). *Dashed lines* represent the membrane curvature average value (C_{mean}). Representative imaging and profiles of 3 independent experiments.

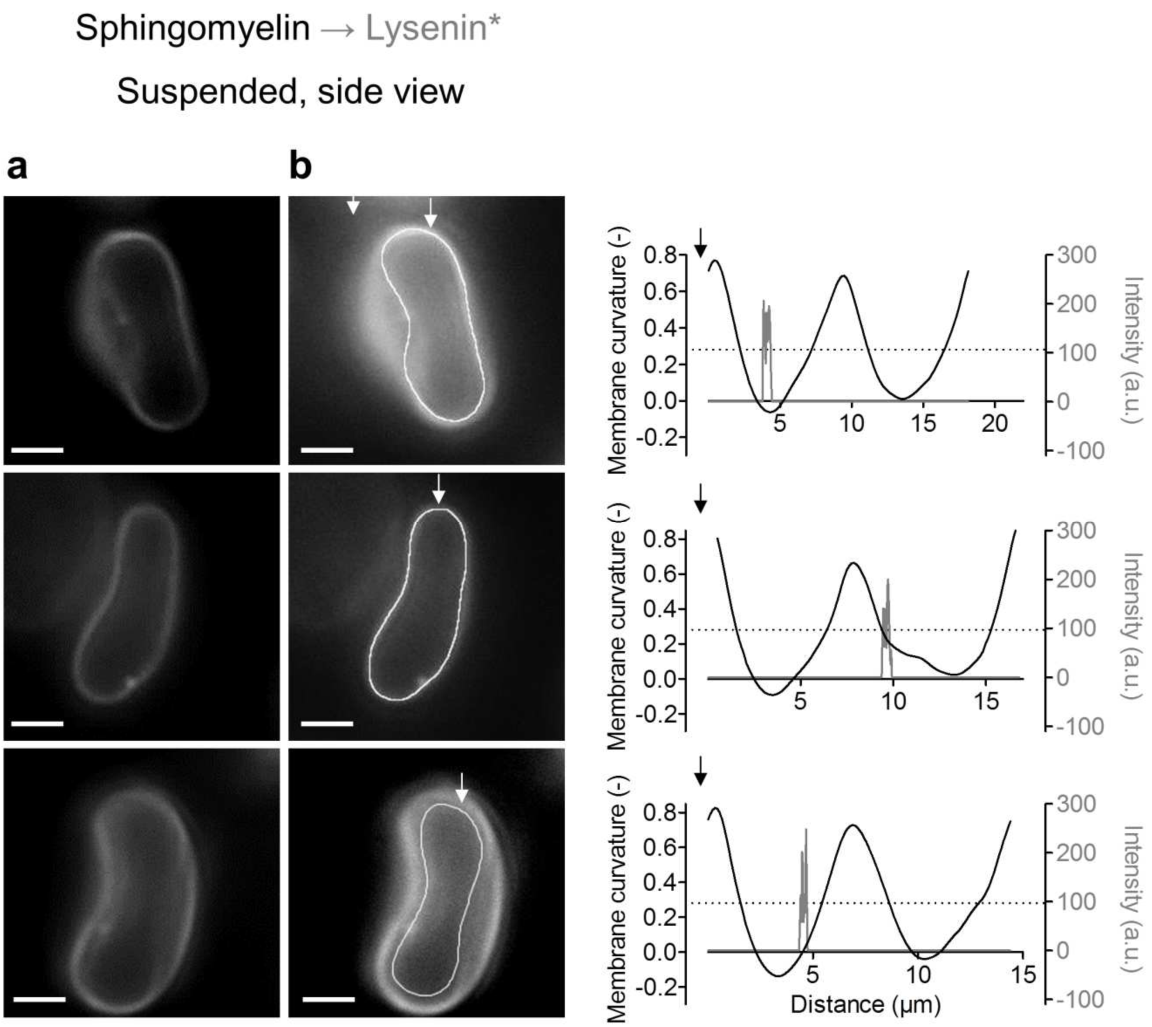

Figure S4 (extension of Fig. 2). Specific association of SM-enriched domains with low curvature areas of the RBC biconcave membrane: gallery of images and profiles. Fresh healthy RBCs were labelled with Lysenin*, then visualized in suspension by vital fluorescence microscopy. (**a**) Representative imaging, s*cale bars* 2 µm. (**b**) Profiles comparing Lysenin* intensity (*grey*; *right panels*) and membrane curvature (*black).* Profiles along paths indicated in *left panel* by *white curved lines* on side-viewed suspended RBCs (counterclockwise from starting point indicated by *white arrows*). *Dashed lines* represent the membrane curvature average value (C_{mean}). Representative imaging and profiles of 3 independent experiments.

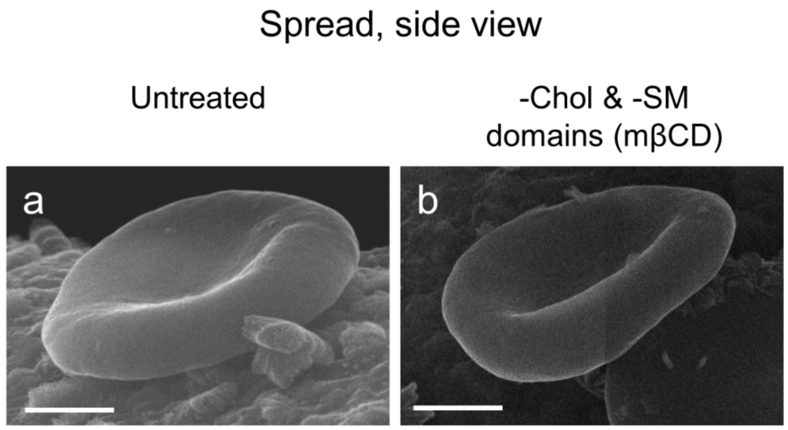

Figure S5 (extension of Fig. 2). Specific association of lipid domains with distinct curvature areas of the RBC biconcave membrane is not involved in the maintenance of this specific shape. Fresh healthy RBCs were either left untreated (a, *untreated*) or incubated with methyl-ß-cyclodextrin (mßCD, b, -*Chol & -SM domains*), then spread and observed in side view by SEM. S*cale bars* 2 µm.

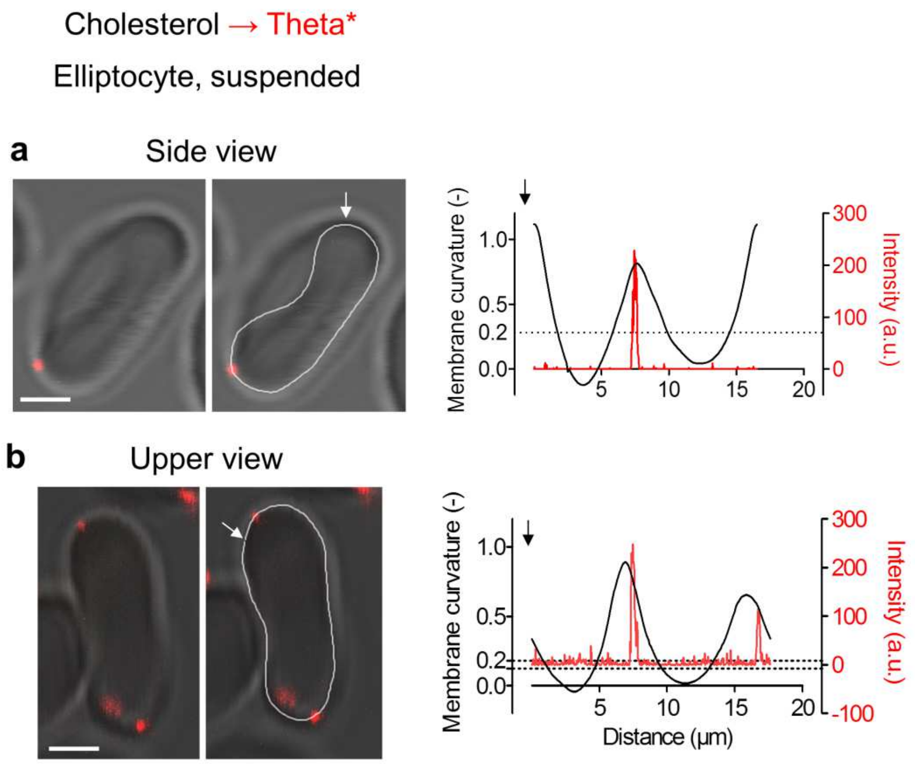

Figure S6 (extension of Fig. 3). Preserved biconcavity in elliptocyte and preferential association of chol-enriched domains with increased curvature areas in the rim of suspended elliptocytes. Fresh elliptocytotic RBCs were labelled in suspension with Theta* then visualized in suspension by vital confocal microscopy either in side view (a) or upper view (b). Representative imaging (*left and central panels)* and profiles comparing Theta* intensity (*red*, *right panels*) and membrane curvature (*black)*. Profiles along paths indicated in *central panel* by *white curved lines* on side- and upper-viewed RBCs in suspension (counterclockwise from starting point indicated by *white arrows*). *Dashed lines* represent either the mean average membrane curvature value (C_{mean}, a) or HC_{min} and HC_{max} (b). Representative imaging and profiles of three independent experiments, s*cale bars* 2 µm.

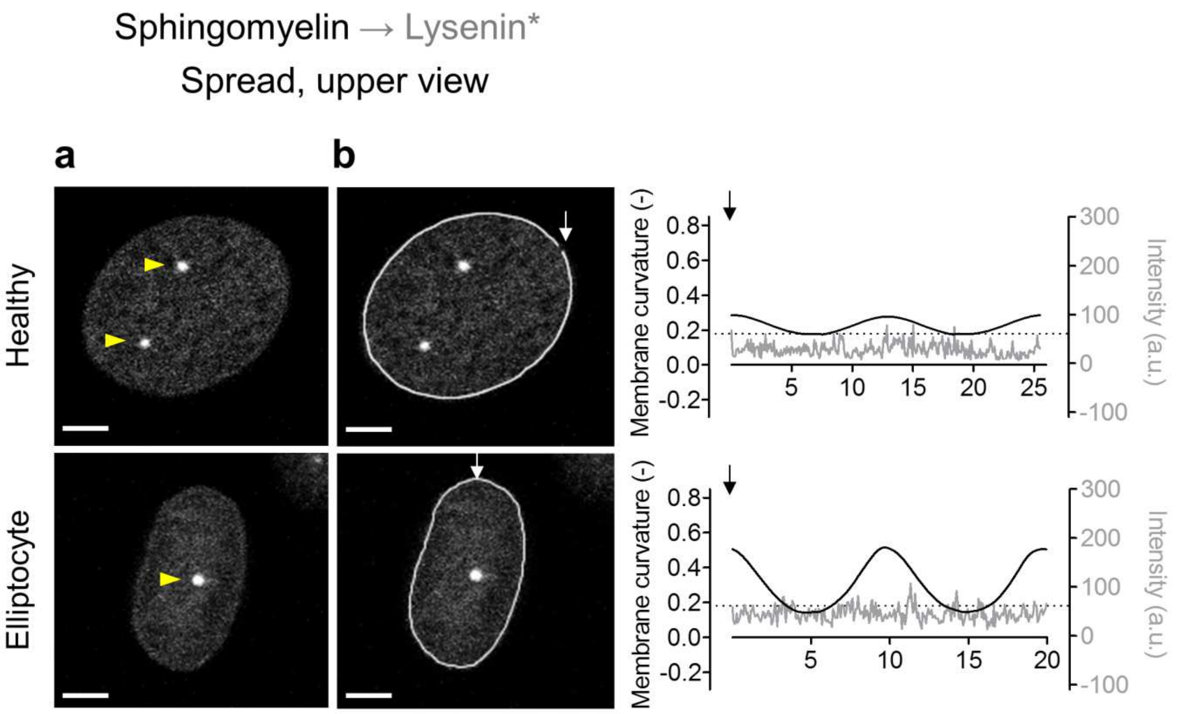

Figure S7 (extension of Fig. 3, 4 & 6). Absence of SM-enrichment in increased curvature areas of the rims of elliptocytes. Fresh healthy (*Healthy*) or elliptocytotic RBCs (*Elliptocyte*) were labelled in suspension with Lysenin*, spread onto PLL-coated coverslip and observed by vital confocal microscopy. (**a**) Representative imaging. Y*ellow arrowheads* indicate domains in LC membrane area, s*cale bars* 2 µm. (**b**) Profiles comparing Lysenin* intensity (*grey*, *right panels*) and membrane curvature (*black)*. Profiles along paths indicated in *left panel* by *white curved lines* (counterclockwise from starting point indicated by *white arrows*). *Dashed lines* represent HC_{max}. Representative imaging and profiles of 2 independent experiments.

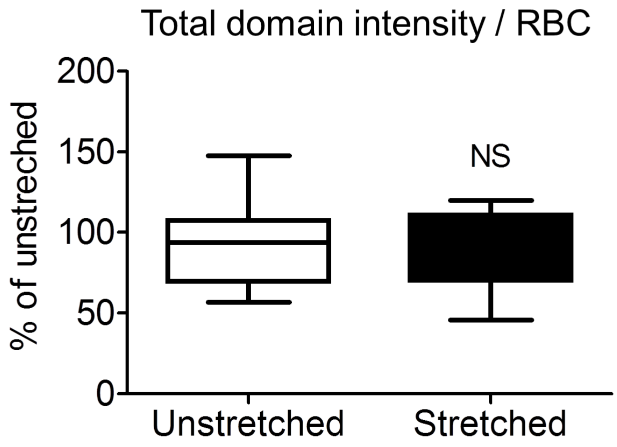

Figure S8 (extension of Fig. 4e). Quantification of total Theta* intensity associated with chol-enriched domains upon RBC stretching. Fresh healthy RBCs were labelled with Theta*, washed and spread onto PLL-coated PDMS chamber and directly visualized by vital fluorescence microscopy before (*unstretched*) and after stretching (*stretched*) as in Fig. 4. Images were analysed for total Theta* intensity of chol-enriched domains by hemi-RBC area (expressed as percentage of unstretched) for unstretched (*open symbols)* and stretched RBCs (*black symbols*). Results are means ± SEM of 13-18 RBCs from two independent experiments. Statistical significance was tested with two-sample t-tests.

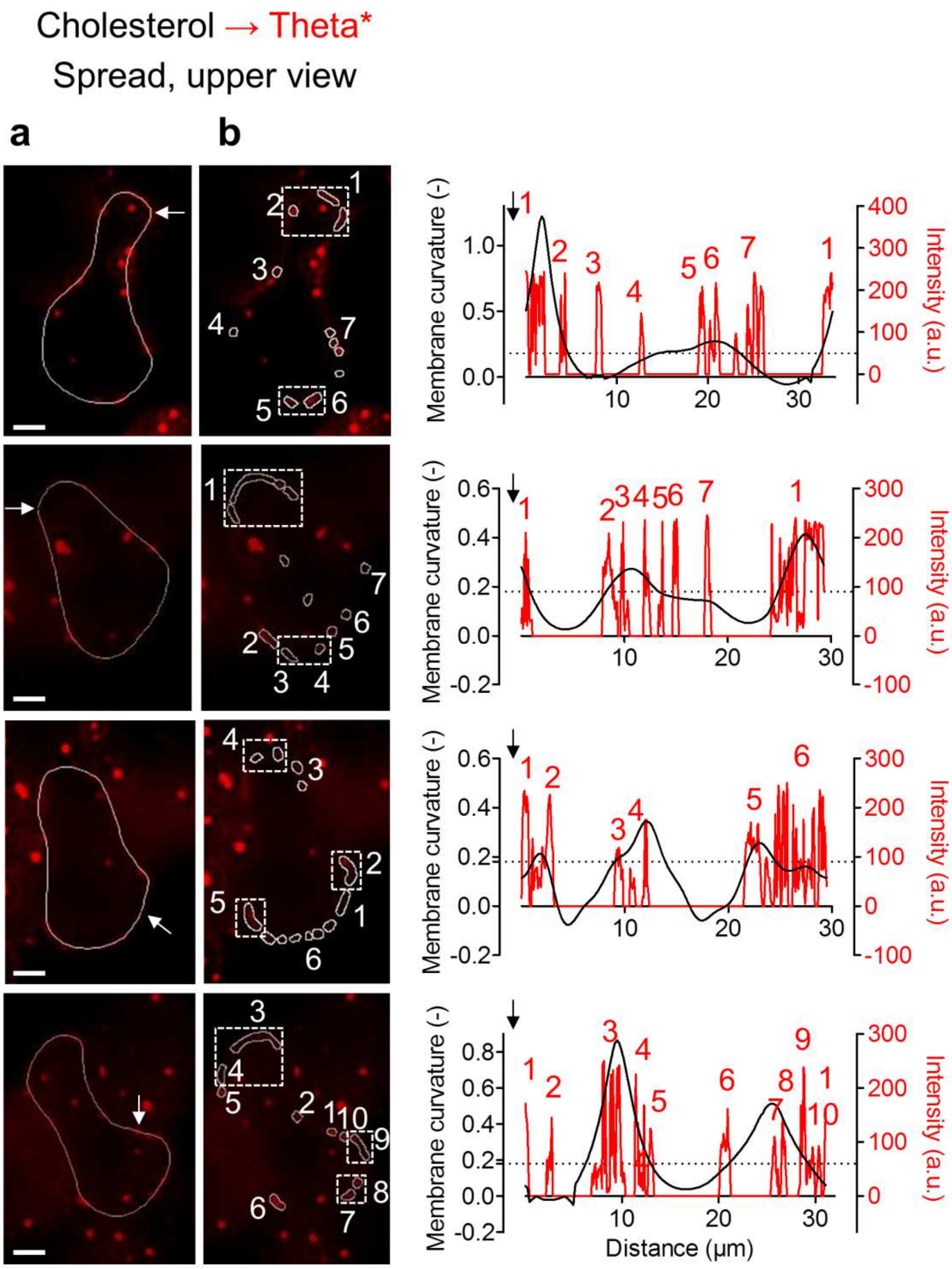

Figure S9 (extension of Fig. 4). Specific recruitment of chol-enriched domains in increased curvature areas of the RBC rims upon stretching: gallery of images and profiles. Fresh healthy RBCs were labelled with Theta*, washed and spread onto PLL-coated PDMS chamber and directly visualized by vital fluorescence microscopy after stretching (*stretched*). (**a**) Representative imaging, s*cale bars* 2 µm. (**b**) Comparison of Theta* intensity (*red*, *right panels*) and membrane curvature (*black*) profiles. Profiles are done along paths indicated in (a) by *white curved lines* starting by *white arrow*; domains (*red numbers*) are labelled with *white numbers* in *left panels*; *dashed lines* represent HC_{min} and HC_{max}. Theta* domains associated with increased curvature (> HC_{max}) are boxed in *left panels*.

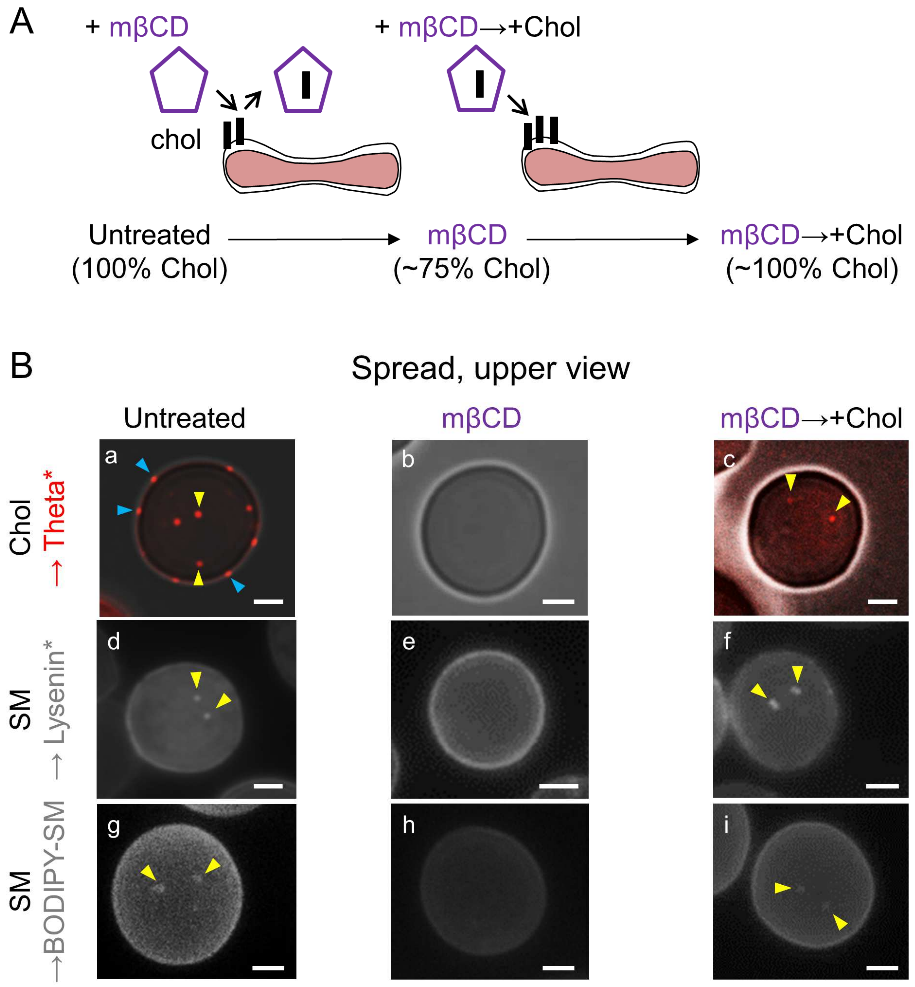

Figure S10 (extension of Fig. 5). Pharmacological approach developed to specifically deplete chol- *vs* SM-enriched domains. (A) Schematic representation of the pharmacological approach. Fresh healthy RBCs were either left untreated (left panel) or incubated with mßCD (to remove ~25% of chol; middle panel) or with mßCD followed by chol repletion (to restore the chol level as in control RBCs; right panel). **(B)** Preferential restoration of SM-enriched domains upon repletion with chol. Fresh healthy RBCs treated as explained in panel A were either labelled with Theta* (a-c) or Lysenin*, spread and observed as in Fig. 1 (d-f); or spread before BODIPY-SM labeling (g-i). Notice that mßCD induces both chol- and SM-enriched domain disappearance (b,e,h) while mßCD followed by chol repletion allows to specifically restore domains in the centre of the RBC (yellow arrowheads at c,f,i) that clearly show SM-enrichment (arrowheads at f,i). Please see text for additional information. *All scale bars*, 2µm.

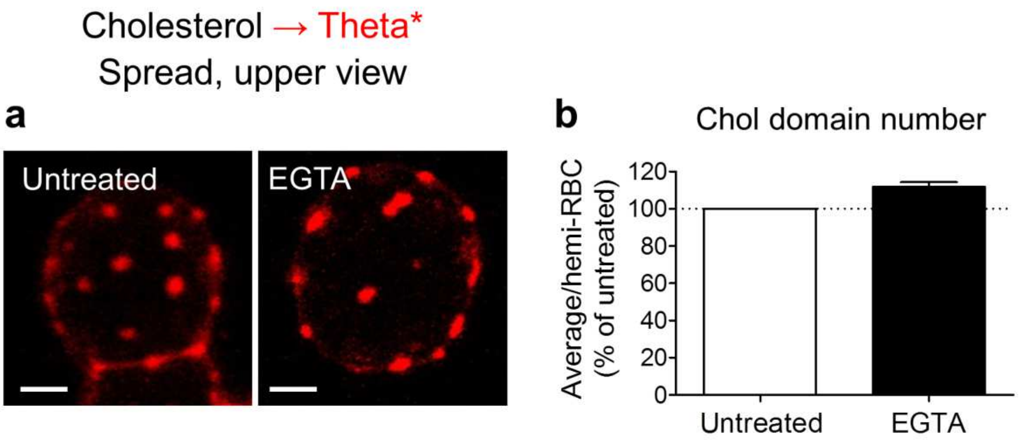

Figure S11 (extension of Fig. 7). Absence of significant increase of chol-enriched domain abundance during calcium efflux. Fresh healthy RBCs were either left untreated (*Untreated & open bars*) or stimulated for Ca^{2+} efflux by incubation with EGTA in Ca^{2+}-free medium (*EGTA & black bars)*. RBCs were then labelled with Theta*, spread onto PLL-coated coverslips and visualized by vital confocal microscopy. (**a**) Representative imaging of two independent experiments, s*cale* bars 2 µm. (**b**) Quantification of domain number (average number by hemi-RBC expressed as a percentage of untreated). Results are means ± SEM of 134-304 RBCs from two independent experiments. Statistical significance was tested with two-sample t-tests.

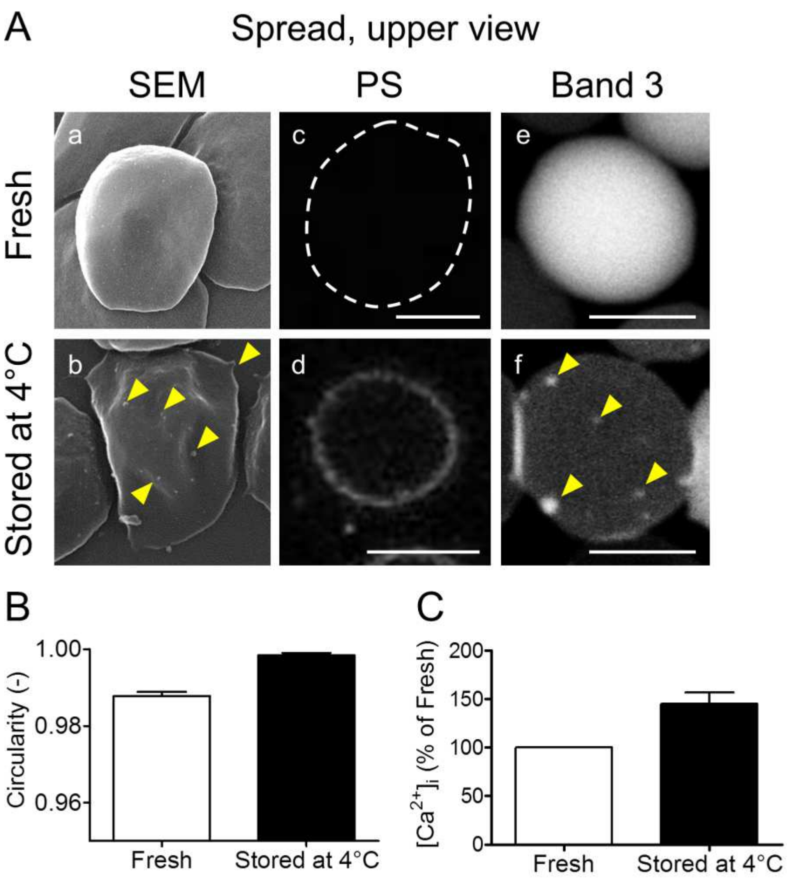

Figure S12 (extension of Fig. 9). Changes to the RBC membrane organization and modulation of RBC circularity and intracellular Ca^{2+} content during blood storage at 4°C. (A) Changes to RBC membrane. Fresh RBCs (*fresh*; a,c,e) or maintained for 2 weeks at 4°C (*stored at 4°C*; b,d,f) were either left unlabelled and analysed by scanning electron microscopy (SEM; a,b), or labelled with Alexa 647-Annexin V (phosphatidylserine [PS] exposure at the outer PM leaflet; c,d) or with Alexa 647-C2-maleimide (Band 3; e,f) and analyzed by vital confocal imaging. Notice microvesicle formation (arrowheads at b), PS translocation to the outer PM leaflet (d) and Band 3 aggregates (arrowheads at f) upon storage at 4°C. *All scale bars*, 5µm. **(B)** RBC circularity for fresh and stored RBCs (by hemi-RBC), measured on transmission images of spread RBCs. **(C)** Intracellular Ca^{2+} content. Fresh RBCs or maintained for 2 weeks at 4°C were measured for $[Ca^{2+}]_i$ thanks to Fluo-4, as at Fig. 6.
